# Genome-wide characterization and comparative expression profiling of *dual-specificity phosphatase* genes in yellow catfish (*Pelteobagrus fulvidraco*) after infection with exogenous *Aeromonas hydrophila*

**DOI:** 10.3389/fimmu.2024.1481696

**Published:** 2024-11-13

**Authors:** Shengtao Guo, Mengsha Zeng, Chenhao Zhang, Yuxin Fan, Miling Ran, Zhaobin Song

**Affiliations:** Key Laboratory of Bio-Resources and Eco-Environment of Ministry of Education, College of Life Sciences, Sichuan University, Chengdu, China

**Keywords:** aquatic pathogens, gene family, molecular evolution, immune response, expression pattern

## Abstract

**Introduction:**

Dual-specificity phosphatases (DUSPs) are crucial regulators in many mammals, managing dephosphorylation and inactivation of mitogen-activated protein kinases (MAPKs) and playing essential roles in immune responses. However, their presence and functions in teleosts, like the yellow catfish (*Pelteobagrus fulvidraco*), remain unexplored.

**Methods:**

In this study, eight *pfDusp* genes (*pfDusp1-7* and *pfDusp10*) were identified in yellow catfish. We characterized their molecular features, conserved protein sequences, and chromosomal localization through genome-wide analyses, and we examined their expression patterns in immune responses.

**Results:**

Our findings reveal two conserved motifs, Leu-Phe-Leu-Gly and Ala-Tyr-Leu-Met, within the DSPc domain of DUSP proteins. The genes were mapped across seven chromosomes without evidence of duplication. Comparative analysis showed high conservation of Dusp genes across vertebrates, with evolutionary analysis suggesting *Dusp3* as a potential intermediate form. *Dusp* transcripts were significantly upregulated in the kidney post-*A. hydrophila* infection.

**Discussion:**

These results suggest the involvement of *Dusp* genes in the immune response of yellow catfish to bacterial pathogens, providing insights into their evolutionary significance and potential applications in aquaculture and molecular breeding.

## 1 Introduction

The innate immune system in various vertebrates is the first line of defense against microbial invasion, in which innate immune cells recognize specific proteins on the cell walls of pathogens to initiate an immune response (1). Individual members of the well-known dual-specificity phosphatase (DUSP) family for the dephosphorylation and inactivation of mitogen-activated protein kinase (MAPK) play regulatory roles in the innate immune system (2). The regulation of these signaling pathways is crucial for mounting an appropriate immune response to external threats. Previous studies reported that a subfamily of DUSPs contains MAPK-binding (MKB) motifs or kinase-interacting motifs (KIMs) that interact with the common docking domain of MAPKs to mediate the interaction between enzymes and substrates (3). DUSPs containing KIMs are generally classified as canonical DUSPs or MAPK phosphatases (MKPs), whereas DUSPs without KIM domains are generally termed atypical DUSPs (4, 5). This functional classification helps in understanding the diverse roles that DUSPs play in different cellular contexts.

Different DUSPs exhibit distinct substrate specificities, subcellular localizations, and functions (6), and typical DUSPs are further divided into three subgroups based on their primary subcellular location (nuclear, cytoplasmic, or both) (7). DUSPs can regulate substrate function with or without phosphatase activity (8). For example, DUSPs control MAPK function by sequestering MAPK in the cytoplasm or nucleus (9). Since both DUSPs and MAPK substrates interact with a target MAPK through its common docking domain, DUSPs can also modulate the signaling of that MAPK by competing with its substrates for binding to the MAPK (10). DUSPs are therefore critical for the regulation of MAPK activity in multiple pathways (11). Thus, DUSP regulation of MAPK pathways influences multiple biological processes, from immune responses to cellular proliferation.

DUSPs are an interesting heterogeneous group of protein phosphatases that dephosphorylate both phosphotyrosine and phosphoserine/phosphothreonine residues within one substrate (12). For the catalysis of each MAPK, the DUSP family catalyzes phosphorylation and dephosphorylation at threonine and tyrosine residues (2). MAPK signaling pathways have been found to regulate numerous biological processes, including cell proliferation, cell death, and cell survival (13). Meanwhile, MAPKs regulate physiological and pathological responses to various extracellular stimuli and environmental stresses in different species (14). The well-known members of the MAPK family include extracellular signal-regulated kinases (ERKs), c-Jun amino-terminal kinases (JNKs), and the p38 subgroup (15). These kinases exhibit dual phosphorylation of threonine and tyrosine residues within the conserved T-X-Y motif (16). Inactivation of a MAPK is hence mediated by serine/threonine phosphatases, tyrosine phosphatases, and DUSP through the dephosphorylation of the threonine residues of the T-X-Y motif within the kinase activation loop (17). This balance between phosphorylation and dephosphorylation is crucial for maintaining cellular homeostasis, especially during immune challenges.

It has been reported that DUSP1 is a key endogenous regulator of the inflammatory response to lipopolysaccharide (LPS) in many mammals (18). In fact, DUSP1 is sensitive to bacterial LPS in macrophages, suggesting that DUSP1 is required for the balance of inflammatory responses in infectious diseases (19). However, previous studies have found that DUSP2 is localized in the nucleus and plays a key role in promoting immune and inflammatory responses that are dependent on the activity of MAPKs with wide expression in the immune tissues (20, 21). DUSP3 is a phosphatase with a relatively small molecular weight, containing a catalytic domain but without a binding domain (22). Studies have shown that DUSP3 may play an important role in the innate immune response, but little is known about its physiological function and corresponding substrates (22). DUSP4 is localized in the nucleoplasm and has been reported to selectively dephosphorylate ERKs and JNKs *in vitro* (23, 24). DUSP5 displays phosphatase activity on ERKs, while the overexpression of *DUSP5* results in the inactivation of ERKs and prevents nuclear translocation (25). DUSP6 preferentially inhibits the catalytic activity of ERKs; however, a reduction of DUSP6 by reactive oxygen species (ROS) results in high ERK activity (26). DUSP7 regulates the activity of ERKs to promote proper chromosome alignment in the process of cell division (27). DUSP10 also plays an important role in mediating innate and adaptive immunity, mainly through the regulation of the JNK signaling pathway in immune effector cells (28). Although these members have been studied extensively in mammals, their roles in non-mammalian species, particularly in fish, remain largely unexplored. Although some members of the DUSP family have been confirmed to play crucial roles in innate immunity, identification and systematic analysis of different members remain rare.

As a native freshwater species with strong market demand, yellow catfish (*Pelteobagrus fulvidraco*) has become one of the most valuable teleost species in the aquaculture industry of China (29). Pathogens such as viruses, bacteria, and fungi infect yellow catfish, leading to dramatic fluctuations in the production of this fish (30–32). Among the pathogenic bacteria, *Aeromonas hydrophila* is one of the most destructive pathogens and has caused large-scale mortality of yellow catfish in recent years (33). Infections by *A. hydrophila* spread rapidly, resulting in severe bacterial sepsis and other damage, and ultimately leading to the death of aquatic animals. Therefore, understanding the immune response mechanisms in yellow catfish is critical for effective disease control and prevention in aquaculture. Since DUSPs are involved in immune responses and infectious diseases in many mammals (34), researchers have conducted numerous studies on the detailed molecular mechanisms of DUSP regulation in bacterial infections and inflammatory responses in recent years (35, 36). Meanwhile, the crystal structures of various DUSPs have been well-analyzed (37, 38).

However, the molecular structures, evolutionary status, and functions of these DUSPs are still unknown in non-mammals, including fish (such as yellow catfish, *P.fulvidraco*). In this study, members of the yellow catfish *Dusp* gene family were identified through a comparative genomics approach based on published genomic data. Before examining the physiological roles of these DUSPs in response to bacterial infection, their conserved domains and spatial structure were predicted and compared with corresponding counterparts in several representative teleost species. Meanwhile, a phylogenetic tree of DUSPs in various vertebrates was constructed for an evolutionary comparison. These comparative analyses highlight the evolutionary conservation and divergence of the DUSP gene family among vertebrates. *In vivo* transcriptional changes of these *Dusp* genes in the kidneys of yellow catfish were compared after being infected by exogenous *A. hydrophila*. The transcriptional data suggest that these genes are actively involved in the immune response to bacterial infection. These new results, derived from both genomic and transcriptomic data, will be valuable for the theoretical understanding and practical application of these immune-related genes.

## 2 Materials and methods

### 2.1 Identification of eight *Dusp* genes from yellow catfish

A chromosome-level assembly of the yellow catfish genome was downloaded from the NCBI database (under accession no. GCF_022655615.1). The raw data were combined with our Illumina transcriptome data (in this study) for genomic analysis. A BLAST search based on protein similarity was performed to identify orthologs of the *pfDusp* genes (*1–7* and *10*) in yellow catfish as we have previously reported (39). These detailed sequences were then aligned with those published sequences for other vertebrates from the NCBI databases.

### 2.2 Analysis of the deduced DUSP protein sequences

Amino acid sequences of these *pf*DUSP (1-7 and 10) proteins from yellow catfish (see more details in **Supplementary Table S1**) were aligned using the ClustalW program (40). These deduced protein sequences were then submitted to SMART (http://smart.embl.de/) to predict their conserved domains. Amino acid number, theoretical isoelectric point, and molecular weight were calculated using ExPASy (41).

### 2.3 Genome-wide localization and genomic synteny analysis of *Dusp* genes in yellow catfish

The chromosomal locations of *pfDusp* genes were extracted from the genome annotation file of yellow catfish (*P. fulvidraco*) and then visualized by using Gene Location Visualize from the GTF/GFF module in Tbtools (42). For the collinearity analysis, the genome sequence and annotation files of a closely related species, darkbarbel catfish (*Pelteobagrus vachelli*; Genebank: GCA_030014155.1) were downloaded from the NCBI, and then MCScanX was applied to analyze the collinearity between the two *Pelteobagrus* fish (E-value was set at 1e-10). Finally, Dual Systeny Plotting was employed in the MCScanX module in Tbtools for visualization (42).

### 2.4 Prediction of protein spatial structures and phylogeny of DUSPs in yellow catfish

In this study, the most recent version of DeepMind AlphaFold2 (https://github.com/deepmind/alphafold) was employed to predict the spatial structures of eight *pf*DUSPs (1-7 and 10), and PyMol 2.5 was used to visualize the data (43). The full-length sequences of DUSP proteins, derived from representative vertebrates, were used to construct a phylogenetic tree, which was established using the neighbor-joining method in the MEGA X software (44). A nonparametric bootstrap analysis was performed to assess the robustness of the topological factors with 1,000 resampling replicates (45).

### 2.5 Fish sample collection and challenge experiment

The yellow catfish (body weight of 32 ± 1.0 g) in this study were purchased from Neijiang Yellow Catfish Breeding Base, Neijiang, Sichuan Province, China. They were delivered to an indoor circulated pond at Neijiang Normal University and fed with commercial feed (protein content at approximately 40%; Huaian Tongwei Feed Co., Ltd., Wuxi, Jiangsu, China) at 28°C every day. The fish were held for 2 weeks, during which time individuals with poor mobility were culled. Additionally, the active and glossy fish from this group were selected for subsequent experiments.

For comparison of the transcriptional changes of the eight *Dusp* genes in response to the exogenous bacterial challenge, six fish were randomly selected for individual intraperitoneal injections of 20 μL of phosphate-buffered saline (PBS, pH 7.2) as the control group. Another group of 12 fish was given individual intraperitoneal injections of 20 μL (2.0 × 10^7^ CFU/mL) of *A. hydrophila* suspension for bacterial challenge. Each group of fish (n=3) was anesthetized with 300 mg/L MS-222 (Sigma-Aldrich, St. Louis, MO, USA) solution 12 or 24 h later and then their kidney tissues were collected for RNA extraction.

All the animal experiments in this study were approved by the ethics committee at the School of Life Sciences of Sichuan University (permit number: SCU221208001), Chengdu, Sichuan Province, China.

### 2.6 RNA extraction and cDNA synthesis for quantitative real-time PCR

The total RNA of the collected kidney tissues was extracted using TRIZOL reagent (Invitrogen, Carlsbad, CA, USA) according to the manufacturer’s protocol. RNA samples were treated with DNase to remove potential genomic DNA contamination. Subsequently, the quality of the isolated RNA was assessed using a NanoDrop 2000 spectrophotometer (Thermo Scientific, Waltham, MA, USA) and agarose gel electrophoresis. RNA samples were reverse transcribed using a Quantscript reverse transcriptase kit (Tiangen Biotechnology, Beijing, China). The obtained cDNA solution was used as the template for subsequent PCR amplification.

The quantitative real-time PCR (qRT-PCR) was performed on a Bio-Rad T100 thermal cycler amplifier (Bio-Rad, Hercules, CA, USA) to measure the transcription levels of eight pf*Dusp* genes. The total volume of each PCR was 20 μL, including 10 μL of SYBR Premix Ex Taq (Tiangen Biotech), 40 ng of cDNA, 0.2 mM of sense and antisense primers, and 7 μL of double-distilled water. These reactions were performed as follows: first at 95°C for 1 min, and then 40 cycles of 95°C for 5 sec, 60°C for 20 sec, and 72°C for 20 sec. Each amplicon was verified as a melting curve (with a single peak) for the target gene. The relative transcription levels of each gene were obtained as previously reported and are expressed as mean ± SEM (n = 3) (46). β-Actin was used as the internal reference gene. The sequences of the primer pairs are provided in **Table 1**.

**Table 1:** Primer pairs for quantitative RT-PCR.

Primer name	Primer sequence (5’-3’)	Amplicon (bp)
*β-actin F*	GGACCAATCAGACGAAGCGA	105
*β-actin R*	TCAGAGTGGCAGCTTAACCG	
*Dusp1 F*	CAAAGGTGGCCGTGTCTTTG	102
*Dusp1 R*	TCGTCCAACTTGACACGGTT	
*Dusp2 F*	AACGAGGCTTTCGAGTTCGT	121
*Dusp2 R*	CCGTGTCCAGAACCGTGTAA	
*Dusp3 F*	GCGGCTTTTACACTTTGCCA	75
*Dusp3 R*	CCACAAATGCGTTCCCGATG	
*Dusp4 F*	GCTCTCGGGAGGATAAGTGC	123
*Dusp4 R*	ATCGATAGCGTGGACGGATG	
*Dusp5 F*	GAGTTGTGTACCGAGGCCAA	92
*Dusp5 R*	ACCGGACCATCCTGAGTGTA	
*Dusp6 F*	CGACTCAAGAAGGGCAACCT	138
*Dusp6 R*	GTCCACGTTCTCGTTCCACT	
*Dusp7 F*	CATGCACAGACTCCGGGAAG	83
*Dusp7 R*	GTTCTGGGAACTCCGTCTGG	
*Dusp10 F*	GCTCTGAGGAAACGCTGCTA	144
*Dusp10 R*	GGAGGGGCATGGCTAATAGG	

### 2.7 Statistical analysis

In this study, all data are expressed as mean ± SEM. Statistical evaluation was performed using repeated measures of one-way ANOVA in GraphPad Prism 9 (GraphPad Software, Boston, MA, USA), and t-tests and Duncan’s multiple range tests were performed. Any difference was considered significant at a statistical level when *p* < 0.05.

## 3 Results

### 3.1 Identification and characterization of *pf*Dusp genes

**Figure 1:**
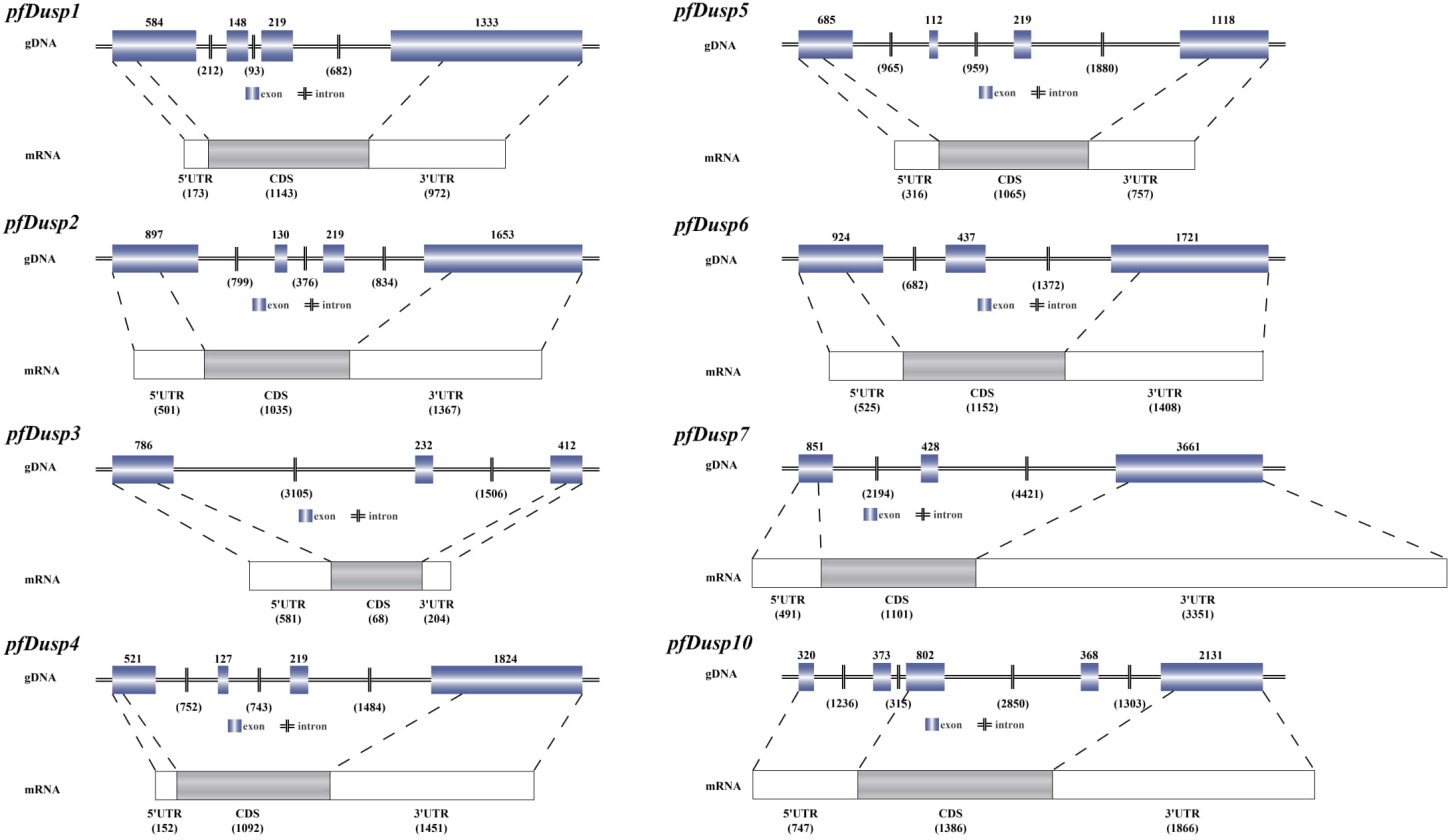
Genomic structures of the eight *pfDusp* genes. Genomic DNA (gDNA) and mRNA sequences were obtained from the NCBI genome database of *P. fulvidraco*. Blue boxes represent exons, and horizontal bars stand for introns. White boxes represent 3’UTR and 5’UTR regions, and gray boxes represent coding sequences (CDS).

In this study, a total of eight *Dusp* homologous genes were identified in the genome data of yellow catfish. According to the nomenclature of *Dusp* genes in other bony fish, the eight genes were separately named *pfDusp1*-*pfDusp7* and *pfDusp10*. The genomic structure of each *Dusp* gene is summarized in **Figure 1**, and, based on the GT-AG rule, the eight *pfDusp* genes (1-7 and 10) contain 3-5 exons and 2-4 introns, respectively (**Figure 1**).

**Table 2** provides a comprehensive overview of the nucleotide and protein sequences of the eight pfDusp genes in yellow catfish. Each gene is accompanied by its nucleotide accession number and displays significant variability in full length, ranging from 1,433 bp for pfDUSP3 to 4,943 bp for pfDUSP7. The open reading frames (ORFs) also differ, with pfDUSP10 having the longest ORF at 1,386 bp, while pfDUSP3 has the shortest at 648 bp. The 5’-UTR and 3’-UTR lengths further illustrate this variability, with pfDUSP6 showing the longest 3’-UTR at 1,408 bp. The predicted protein lengths range from 215 amino acids (aa) for pfDUSP3 to 461 aa for pfDUSP10. The molecular weights of these proteins vary from 24.3 kDa for pfDUSP3 to 51.3 kDa for pfDUSP10, and their theoretical isoelectric points (pI) span from 4.97 for pfDUSP6 to 9.15 for pfDUSP3, indicating a range of potential functions and stability under different pH conditions. Notably, all the pfDUSP proteins lack signal peptides and transmembrane domains, suggesting that they are likely cytoplasmic proteins that may play important roles in cellular signaling pathways rather than membrane-bound functions. Overall, the table highlights the structural diversity of the pfDusp proteins, which may be crucial for their varied biological functions in yellow catfish.

**Table 2:** Nucleotide/protein sequences of the eight *pfDusp* genes in yellow catfish.

Gene Name	Nucleotide Accession Number	Full length (bp)	ORF (bp)	5’-UTR (bp)	3’-UTR (bp)	Protein Accession Number	Predicted protein (aa)	Molecular Weight (kDa)	Theoretical pI	Signal peptide	Transmembrane
*pfDUSP1*	XM_027154738	2,288	1,143	173	972	XP_027010539	380	41.6	6.51	NO	NO
*pfDUSP2*	XM_027151906	2,903	1,035	501	1,367	XP_027007707.1	344	38.3	6.08	NO	NO
*pfDUSP3*	XM_027137744	1,433	648	581	204	XP_026993545.1	215	24.3	9.15	NO	NO
*pfDUSP4*	XM_027139834	2,695	1,092	152	1,451	XP_026995635.1	363	39.8	6.09	NO	NO
*pfDUSP5*	XM_027168730	2,138	1,065	316	757	XP_027024531.1	354	39.7	6.92	NO	NO
*pfDUSP6*	XM_027170607	3,085	1,152	525	1,408	XP_027026408.1	383	42.6	4.97	NO	NO
*pfDUSP7*	XM_027136543	4,943	1,101	491	3,351	XP_026992344	366	40.6	5.35	NO	NO
*pfDUSP10*	XM_027147557	3,999	1,386	747	1,866	XP_027003358	461	51.3	6.08	NO	NO

UTR, untranslated region; ORF, open reading frame; aa, amino acid.

**Figure 2:**
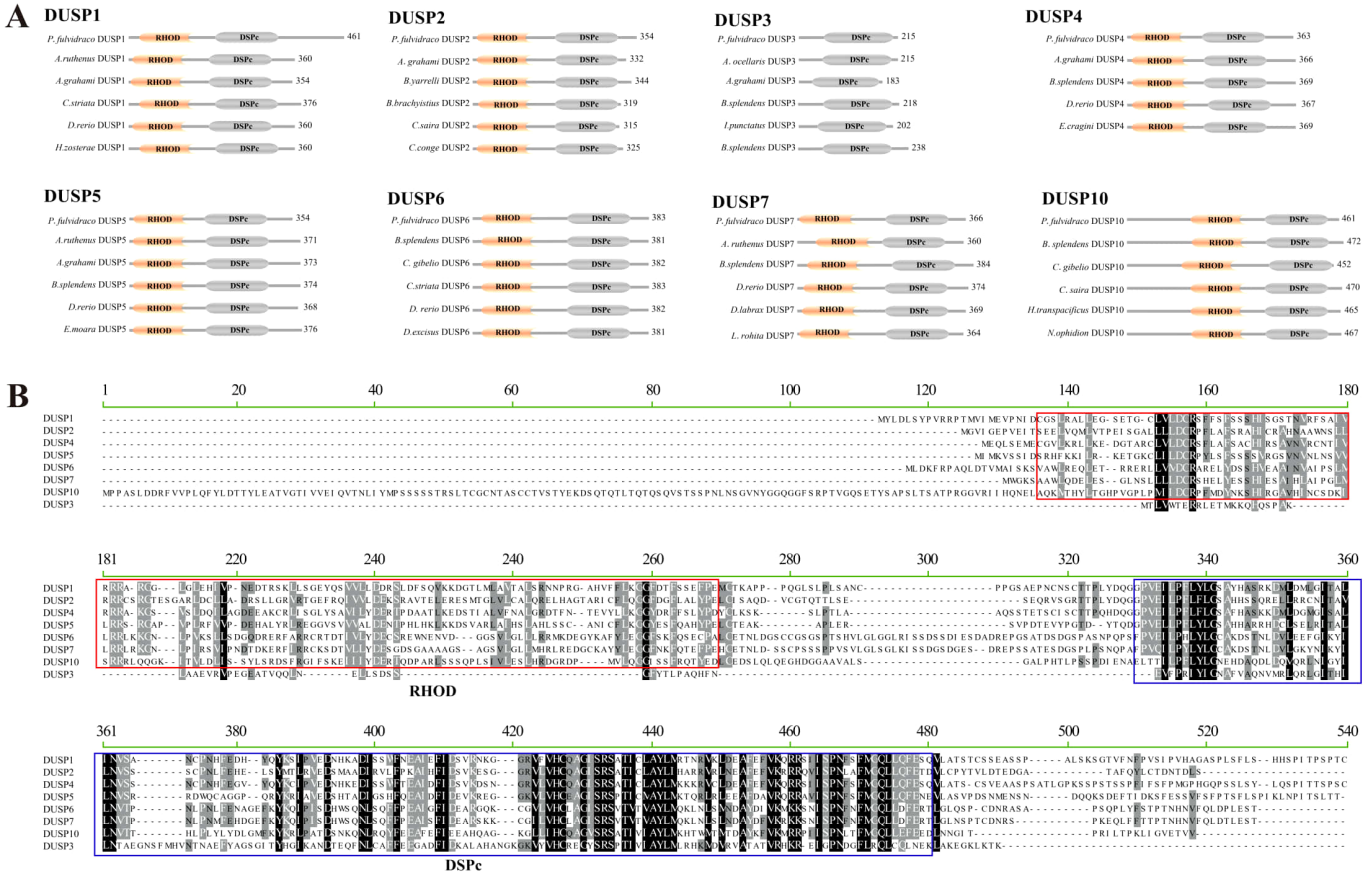
Conserved domains in the eight DUSP proteins. **(A)** Schematic representation of the eight DUSP (1-7 and 10) protein architectures in yellow catfish and other teleost species. Light yellow areas represent RHOD domains, and gray areas represent DSPc domains. **(B)** Multiple sequence alignment of yellow catfish DUSP (1-7 and 10) proteins using MEGA-X. The solid red box represents the RHOD domain, and the solid green box represents the DSPc domain.

Additionally, predictions of conserved domains in the 19 representative fish in this study showed that in yellow catfish, eight DUSPs shared DSPc and rhodanese homology (RHOD) domains, except for DUSP3 with only one DSPc domain (**Figure 2**). The functional domain composition of these DUSPs from the other 18 vertebrate species is completely consistent with that of the yellow catfish. Multiple sequence alignment indicated that there are two common tetramers (Leu-Phe-Leu-Gly and Ala-Tyr-Leu-Met) in the DSPc domain, which appears to be highly conserved in all the identified *pf*DUSPs. The alignment also revealed high sequence conservation in the DSPc domain.

### 3.2 Synteny comparisons of *Dusp* genes in representative vertebrates

**Figure 3:**
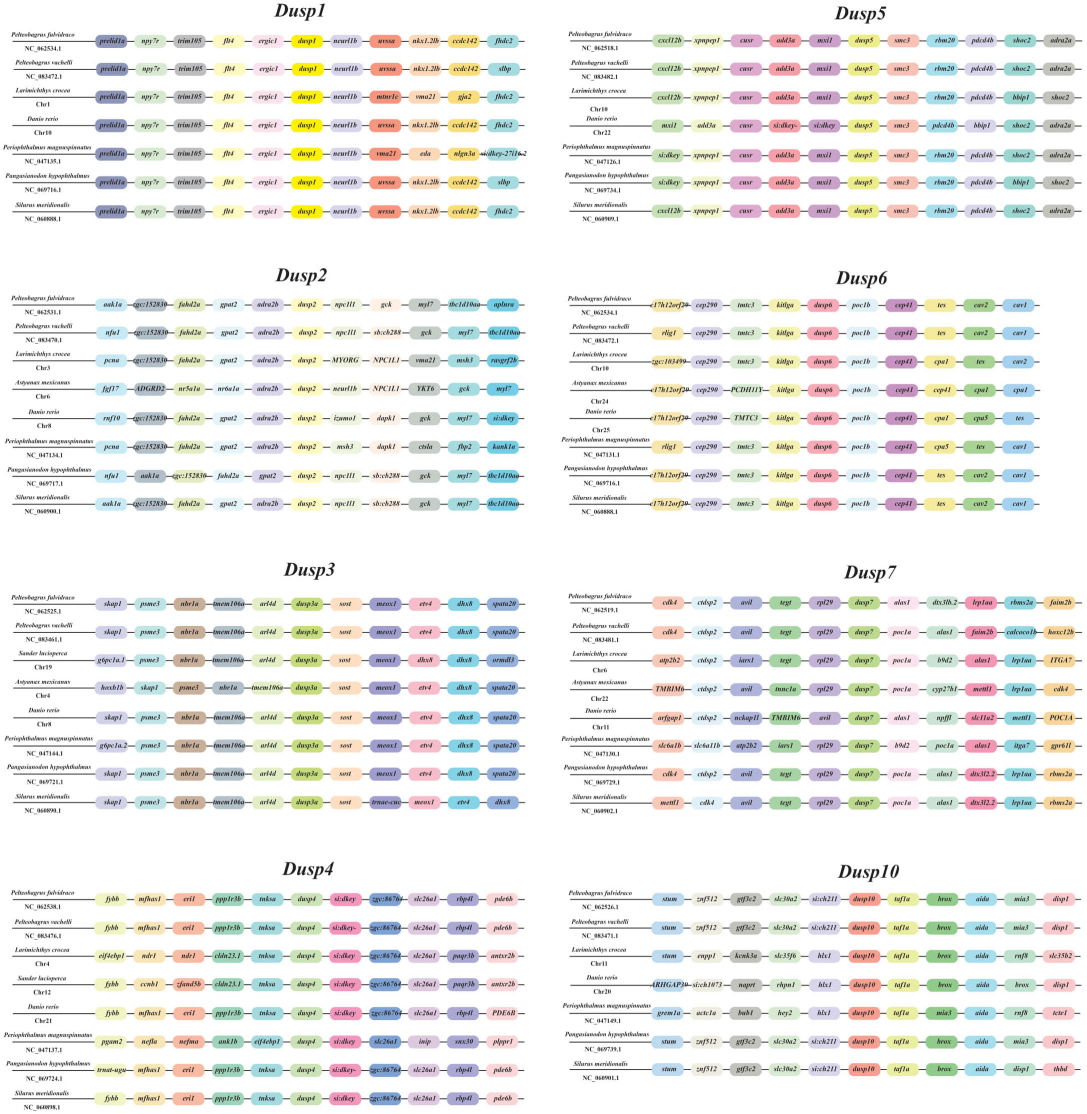
Synteny comparisons of the *Dusp* genes in representative vertebrates. Colored blocks represent different genes. Solid lines mark intergenic regions. Each vertebrate species and its NCBI accession number in genome resources is provided in each row.

In order to verify the accuracy of the *Dusp* genes (1-7 and 10) in teleosts, a comparative genomic synteny analysis among various vertebrates was performed. Our results showed that all the examined species contain the *Dusp*1-7 and *10* genes, and the order of *Dusp* genes and their neighboring genes was generally well conserved in the genomes of different teleost species (**Figure 3**). Moreover, eight conserved gene clusters with each *Dusp* (*flt4*-*ergic1*-*DUSP1*-*neurl1b*-*uvssa*, *gpat2*-*adra2b*-*DUSP2*-*npc1L1*, *tmem106a*-*arl4d*-*DUSP3*-*sost*-*meox1*, *ppp1r3b*-*tnksa*-*DUSP4*-*SI:dkey*-*slc26a1*, *add3a*-*mxil*-*DUSP5*-*smc3*-*rbm20*, *tmtc2*-*kitlga*-*DUSP6*-*poc1b*-*cep41*, *tegt*-*rpl29*-*DUSP7*-*alas1*, and *slc30a2*- *DUSP10*-*taf1a*-*box*) were identified in these teleost species, respectively. In conclusion, the gene orders of the *Dusp* clusters (*1-7* and *10*) are highly conserved in teleost species.

### 3.3 Chromosomal location of the *Dusp* genes (*1-7* and *10*) and cross-species collinearity comparison at the chromosomal level

**Figure 4:**
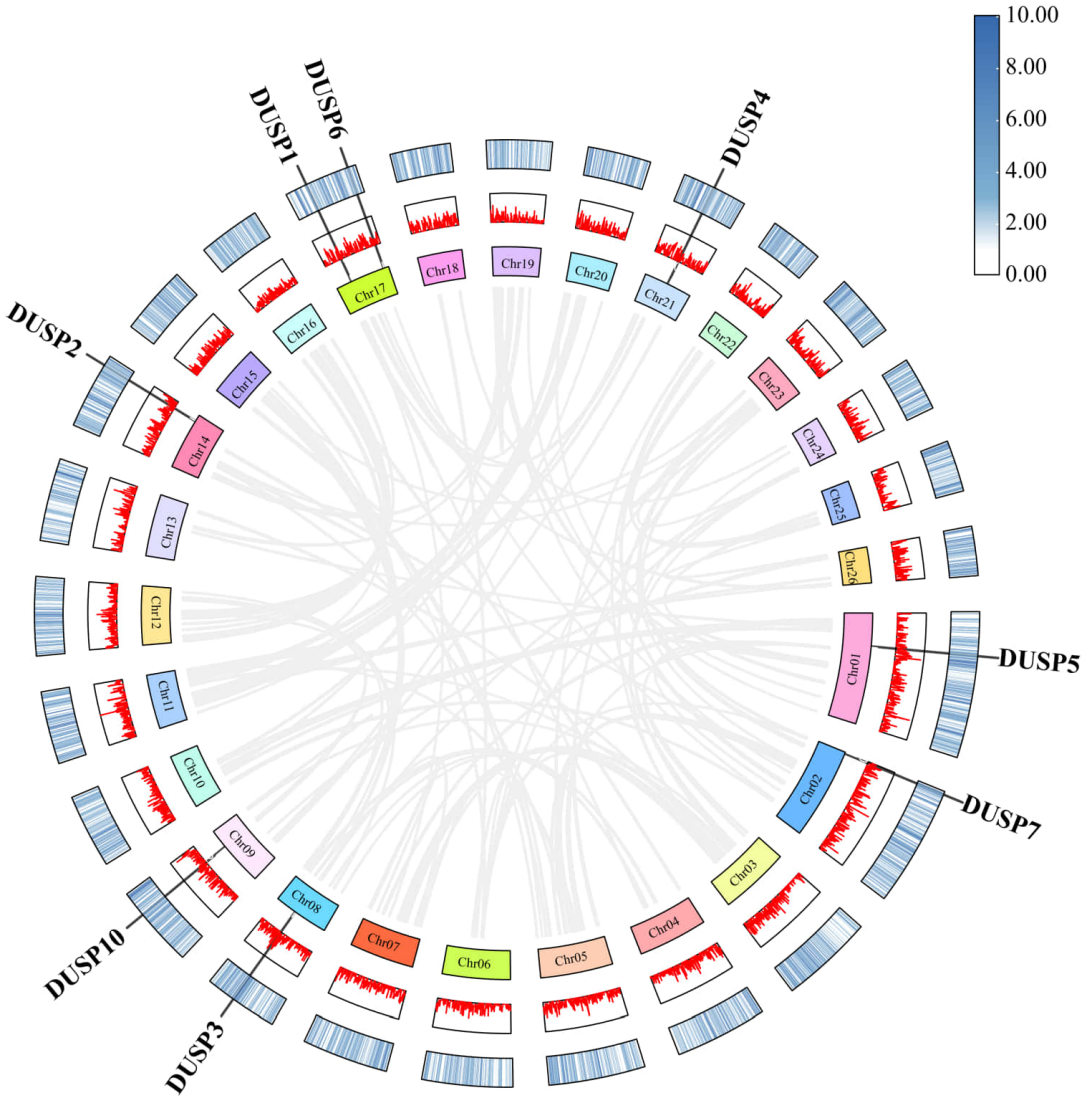
Chromosomal locations and duplication modes among *Dusp* genes (*1-7* and *10*) in yellow catfish. Gray lines represent the syntenic blocks across the genome. The connections within the plot represent potential interactions or synteny among the DUSP genes across the genome. The figure highlights the distribution of the DUSP gene family in yellow catfish.

The chromosomal location of the eight *Dusp* genes was predicted using the NCBI *P. fulvidraco* genome data. Our results showed that *Dusp5*, *Dusp7*, *Dusp3*, *Dusp10*, and *Dusp2* are respectively located on Chr1, 2, 8, 9, and 14 (**Figure 4**). Furthermore, the *Dusp1* and *Dusp6* genes are located on the same Chr17, and *Dusp4* is located on Chr21 (**Figure 4**). It appears that these *Dusp* genes are somehow distributed genome-wide.

**Figure 5:**
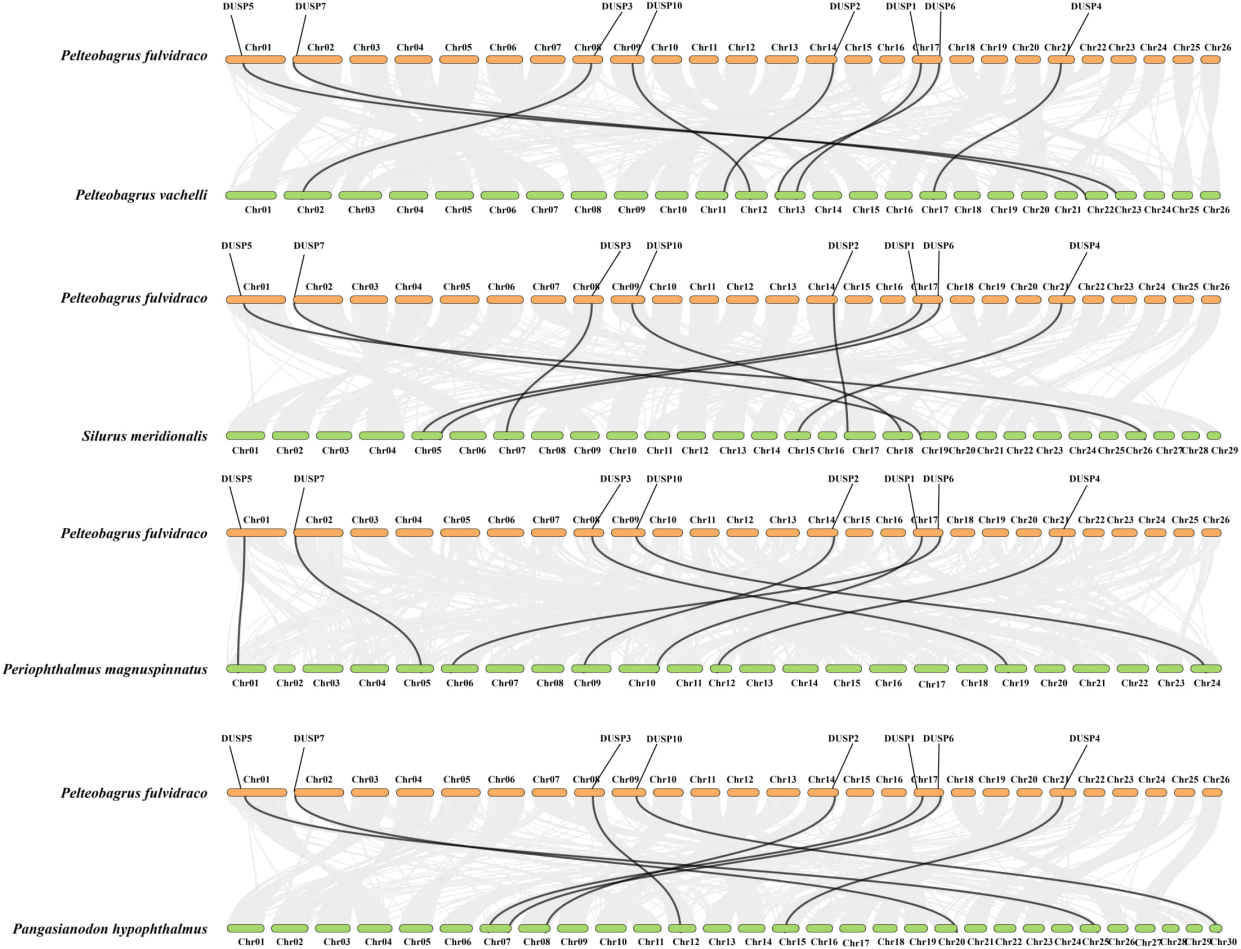
Collinearity of the eight *Dusp* genes between yellow catfish and the other five representative teleost species. Black lines denote the collinearity of *Dusp* genes (*1-7* and *10*) between yellow catfish and other teleosts, while gray lines indicate collinearity between the genomes of yellow catfish and other teleosts. Different colored boxes represent the chromosomes of the corresponding species.

The similarity of nucleotide sequences of *Dusp* genes (*1-7* and *10*) in the yellow catfish was compared to their counterparts from other species. Corresponding collinearity maps of yellow catfish *P. fulvidraco* along with four other teleost species (including darkbarbel catfish *P. vachelli*, Southern catfish *Silurus meridionalis*, giant-fin mudskipper *Periophthalmus magnuspinnatus*, and iridescent shark *Pangasianodon hypophthalmus*) were constructed, respectively (**Figure 5**). There are eight pairs of homologous *Dusp* genes, indicating a high homology in the *Dusp* gene family in different species. In general, *Dusp* genes are highly conserved among the various teleost species, and the *Dusp* genes in yellow catfish are closer to those in darkbarbel catfish due to both being relatives in the same genus.

### 3.4 Spatial structures and phylogeny of various DUSPs

**Figure 6:**
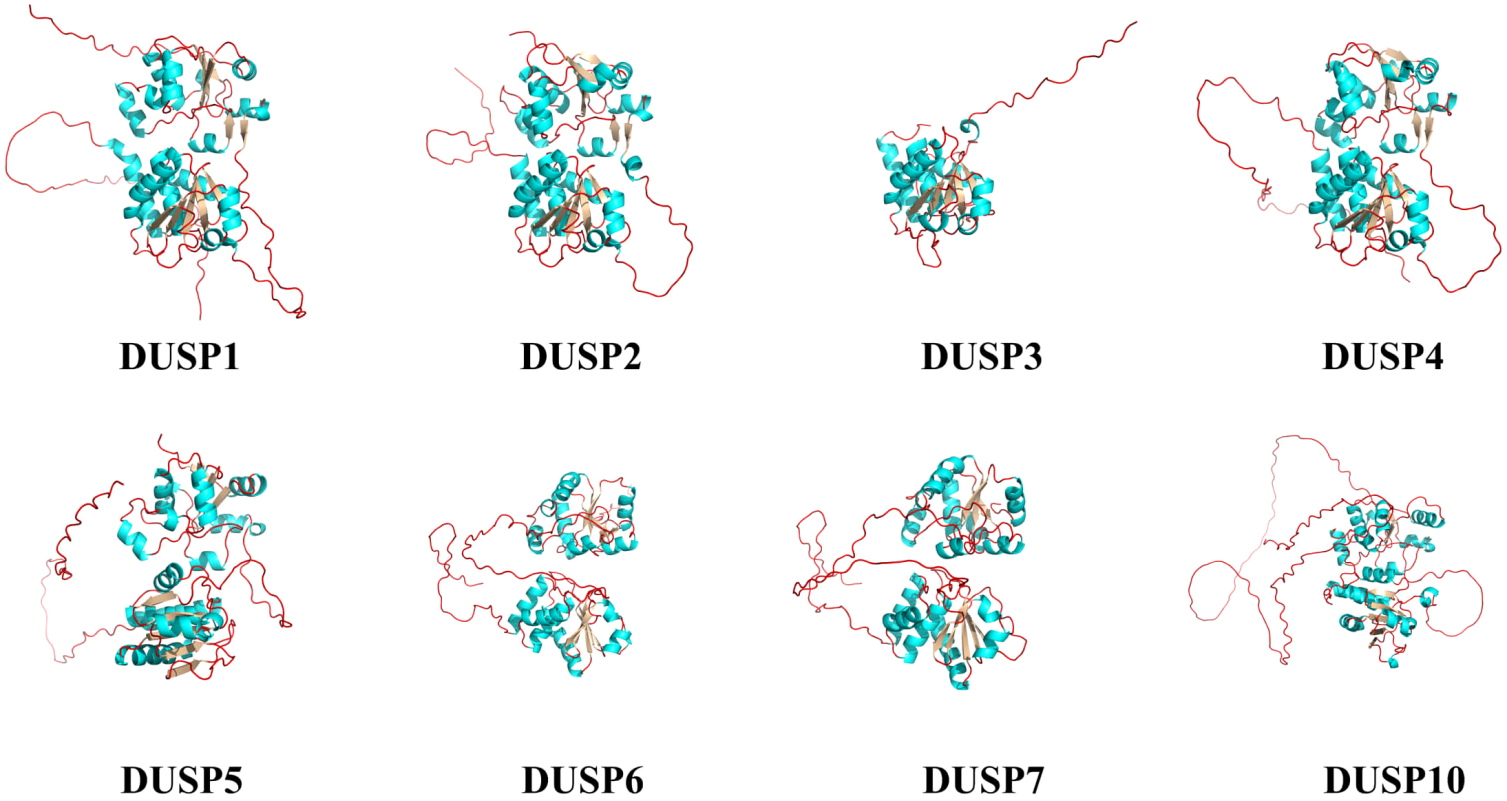
Spatial structures of the eight *pf*DUSP proteins. Green areas represent α-helices, silver areas represent β-sheets, and red lines represent random coils.

To illustrate the various DUSPs in this study, the protein spatial structures of *pf*DUSPs (1-7 and 10) were predicted. We observed that the majority of DUSPs are in the dimer form, although DUSP3 is in a monomeric form (**Figure 6**). All DUSPs share a conserved DSPc functional domain, which is composed of five β-sheets and five α-helices intertwined with each other.

**Figure 7:**
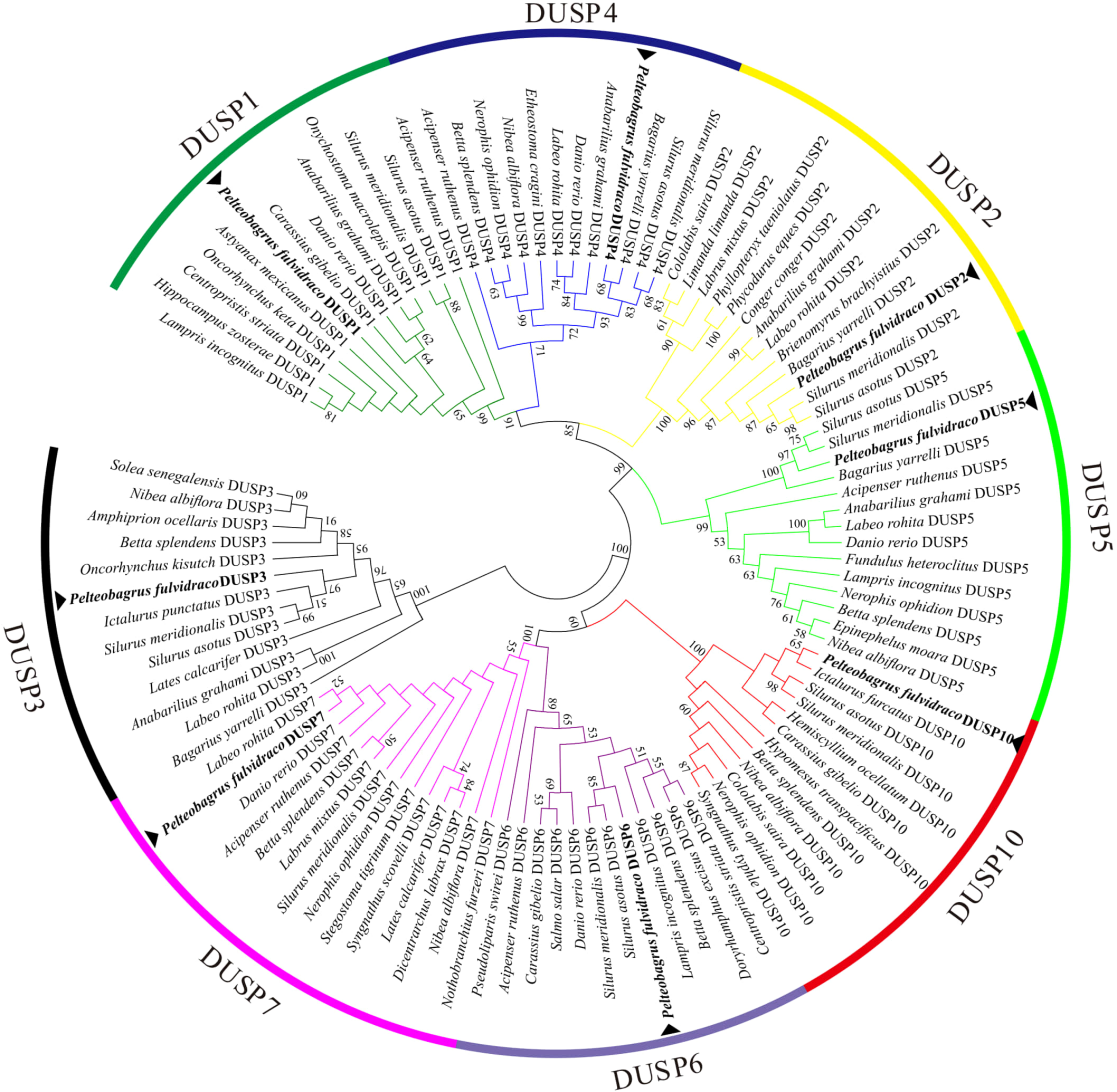
A phylogenetic tree of DUSPs in representative vertebrates. The tree was constructed using the neighbor-joining method. The numbers at the nodes represent bootstrap percentages. DUSPs of *P. fulvidraco* are marked with a ▴.

For a better understanding of the evolutionary relationship of *Dusp* genes in representative vertebrates, a phylogenetic tree based on the amino acid sequences of eight *pf*DUSPs and 95 sequences from other vertebrates was constructed using the neighbor-joining method. As shown in **Figure 7**, 103 DUSPs were divided into eight distinct subfamilies (DUSP1-7 and 10). The phylogenetic topology supports the high conservation of these DUSPs in various vertebrates.

### 3.5 Transcription of *Dusp* genes in yellow catfish after being infected by *A. hydrophila*

**Figure 8:**
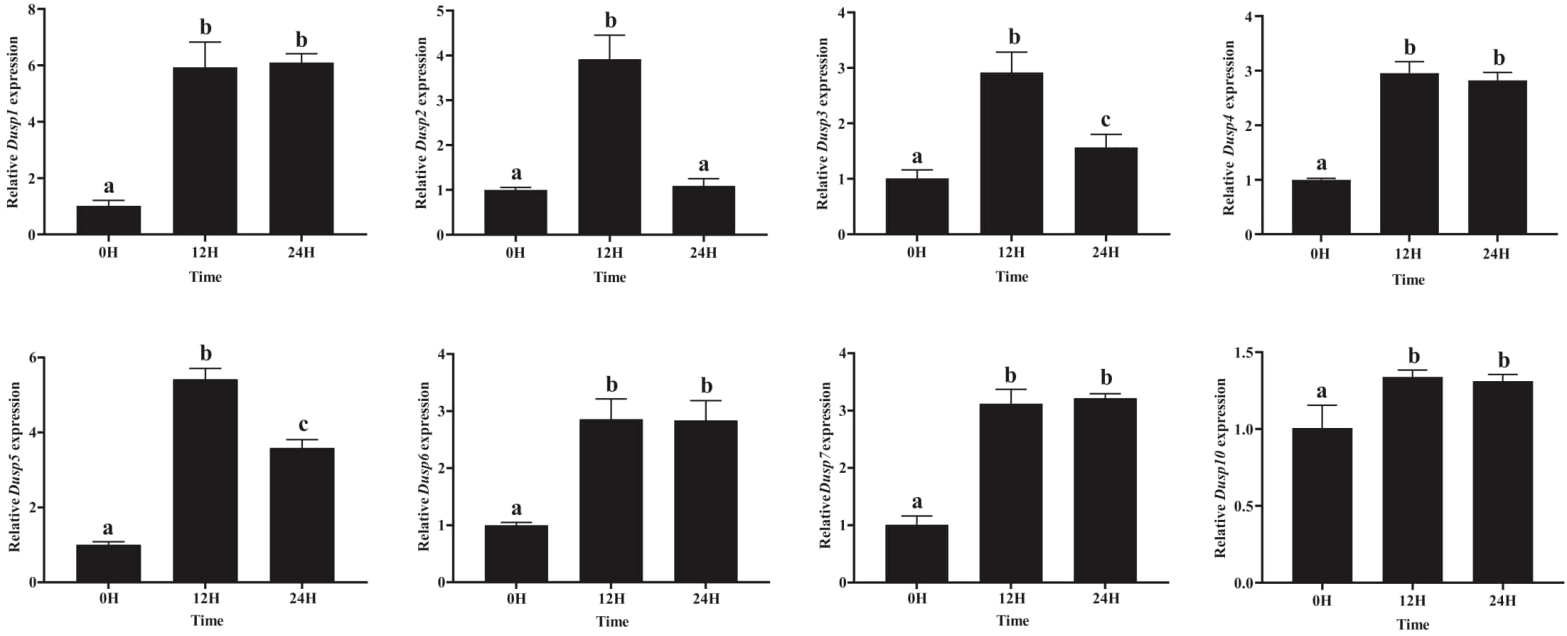
Comparative transcription profiles of the eight *Dusp* genes in the kidneys of yellow catfish. The relative expression levels of the Dusp gene family (Dusp1, Dusp2, Dusp3, Dusp4, Dusp5, Dusp6, Dusp7, and Dusp10) in yellow catfish at different time points (0 h, 12 h, and 24 h) following bacterial infection. Expression levels were measured using qRT-PCR, and values were normalized to the control (0H). Statistical analysis was performed, and significant differences between time points are indicated by different letters (a–c), with the same letters indicating no significant difference (*P* < 0.05).

Since the kidney is a key immune organ in different fish species, the transcription profiles of *Dusp* genes in the kidneys of yellow catfish were determined. In brief, the transcription of eight *Dusp* genes was significantly upregulated in the kidneys at 12 h after the *A. hydrophila* infection (**Figure 8**), indicating that *pfDUSP*s respond to exogenous *A. hydrophila* infection.

The eight *pfDusp* genes (*1-7* and *10*) exhibited their highest transcription levels at 12 h after the *A. hydrophila* infection (**Figure 8**). Meanwhile, *pf*Dusp 1, 4, 6, 7, and 10 maintained the same transcription levels at 24 h after the *A. hydrophila* infection, while *pfDUSP*2, 3, and 5 decreased at 24 h compared with 12 h. After 24 h of infection with *A.hydrophila*, except for the similar transcription level of *Dusp2*, the transcription levels of the remaining seven *Dusp* genes (*1*, *3-7*, and *10)* were significantly higher than the control group (**Figure 8**).

## 4 Discussion

Mammals have evolved a complex network of regulatory mechanisms within their immune systems to balance defense against pathogens while protecting the host from excessive damage (47). Kinases and phosphatases involved in the MAPK signaling pathways are key regulators in the orderly action of pro- and anti-inflammatory processes (48). DUSPs belong to a family of proteins responsible for the dephosphorylation of threonine/serine and tyrosine residues on different substrates (49). This regulation of phosphorylation is crucial in maintaining cellular homeostasis during immune responses. Regulation of the expression and catalytic activity of DUSP family members in different cells and tissues controls MAPK intensity and duration, thereby determining the related physiological response(s) (50). However, the majority of studies on the *Dusp* genes have focused on higher mammals. A few reports have identified this gene family in teleost species, but not from a genome-wide perspective, which limits a further understanding of the evolution and function of *Dusp* genes in fish species. As a result, the study of these genes in fish could offer new insights into their immune roles in teleosts and provide a broader understanding of DUSP evolution. This work is a systematic study of the *Dusp* genes in yellow catfish at the whole-genome level. Eight *Dusp* homologous genes with conserved functional domains were identified in the yellow catfish genome, and the sequence characterization and molecular phylogeny of these genes were determined. Finally, the transcriptional changes of eight *Dusp* genes in the kidneys of yellow catfish after infection with exogenous *A. hydrophila* were measured and analyzed. These results further support the potential role of Dusp genes in immune responses, especially in aquatic organisms, where bacterial infections are prevalent. This study provides new insights into the evolution and expression patterns of *Dusp* genes in a representative teleost fish, and it is of value for elucidating the function of *Dusp* genes in innate immunity.

Our molecular characterization of the *Dusp* gene family members’ results indicated that the *pfDusp* genes are highly conserved in various vertebrates, implying that *Dusp* genes are critical, with crucial functions in different organisms. This conservation suggests a possible universal role for DUSPs across species in immune regulation. Moreover, all *pfDusp* genes contain multiple exons and introns and share the same DSPc domain, which is similar to the structural characteristics of *Dusp* gene family members in other species (4, 10). This domain is known for its catalytic activity in dephosphorylation, further indicating its critical role in the immune response. In fact, our results of the spatial structure analysis of DUSPs showed that the DSPc domains of different DUSP proteins are similar, while there are obvious differences in the amino acid sequences and protein spatial conformation of the RHOD domain. Based on the structural characteristics of the eight homologous genes, we speculate that eight *pf*DUSPs may perform various regulatory functions in yellow catfish. These structural differences may imply functional diversification, suggesting that different DUSPs may target different MAPK pathways or other substrates.

Our gene synteny analysis showed that eight conserved clusters are widely distributed in the genomes of almost all the examined species and that *Dusp* genes are highly conserved during the process of evolution, suggesting that they may play a similar immune role in different teleost fish. The presence of these conserved gene clusters across species underscores their evolutionary importance in immune functions. It is worth noting that none of the eight homologous genes exhibited gene transfer events, which is consistent with the chromosomal location of *Dusp* homologous genes in other fish species. Furthermore, the DSPc domains in the *pf*DUSP proteins share the “Leu-Phe-Leu-Gly” and “Ala-Tyr-Leu-Met” residues, further confirming that DUSPs are evolutionarily conserved. These shared residues may be critical for maintaining the structural integrity and catalytic efficiency of the DSPc domain.

Reliable phylogenetic trees provide a better understanding of the evolutionary history and evolutionary origins of genes and species (51). In the functional domain prediction of 19 teleost DUSPs, we observed that DUSP3 only consists of a single DSPc domain, while the remaining seven DUSPs (DUSP1, 2, 4-7 and 10) contain two functional domains: RHOD and DSPc. Usually, the catalytic functions of DUSPs are realized by the catalytic domain DSPc (52). This suggests that DUSP3 may have a more specialized role compared to the other DUSPs, which have broader substrate recognition capabilities. Rhodocyanase homologous domains are ubiquitous in vertebrate genomes, while the specific functions of these domains remain largely enigmatic (53). Meanwhile, DUSP3 was in a separate branch of the phylogenetic tree, but the remaining seven DUSPs (DUSP1, 2, 3-7, and 10) were clustered into a large branch, suggesting that DUSP3 may be an intermediate transitional form of the *Dusp* gene during the evolution from ancient DUSPs. This phylogenetic placement highlights the possible unique evolutionary trajectory of DUSP3 among DUSP family members.

Fish are the largest group of vertebrates that exhibit both innate and adaptive immunity (54). In various teleost species, the kidneys serve as a major site of hematopoiesis, generating immune cells such as macrophages, lymphocytes, and granulocytes. These cells are critical in both innate and adaptive immune responses, helping to recognize and eliminate pathogens (55). Furthermore, the kidneys are the largest immune organ in fish, and they play an indispensable role in innate immunity (56, 57). Given its central role in fish immunity, studying gene expression in the kidneys can provide insight into immune responses at the organ level. To date, there have been no reports on the immune response of the *Dusp* family in fish after being infected by *A. hydrophila*. To study whether *pfDUSP* genes play a role in antibacterial immune responses, we measured the transcription of individual genes after an *A.hydrophila* infection, which showed that *pfDusp* (1-7 and 10) genes were significantly upregulated in the kidneys after the bacterial infection. This upregulation suggests an active role of these genes in mediating the immune response during bacterial invasion. Similarly, the mRNA levels of *poDusp1* and *poDusp6* in Japanese flounder *Paralichthys olivaceus* and *omDusp1*, *omDusp2*, and *omDusp4* in rainbow trout *Oncorhynchus mykiss* were previously reported to be significantly upregulated after stimulation by exogenous bacteria, such as *Edwardsiella tarda*, *Vibrio anguillarum*, and *Streptococcus iniae* (10, 58–60). Such consistent transcriptional responses across species suggest that *Dusp* genes play a crucial role in the fish immune system’s response to bacterial pathogens. These data demonstrate that infections with different bacterial pathogens indeed induce transcriptional variations in *Dusp* genes. Thus, *Dusp* genes appear to be highly responsive to bacterial infections in various fish species, making them promising targets for future functional studies. Despite these findings, the specific molecular mechanisms by which the *pfDusp* gene family contributes to the immune response remain unknown. Further investigations, such as functional studies using gene knockouts or proteomic approaches, are necessary to elucidate the precise pathways and interactions involved. These studies may provide critical insights into how *Dusp* genes mediate immune defense, paving the way for targeted therapeutic strategies against bacterial infections in aquaculture.

## 5 Conclusion

A total of eight *Dusp* homologous genes, named *pfDusp1*, *pfDusp2*, *pfDusp3*, *pfDusp4*, *pfDusp5*, *pfDusp6*, *pfDusp7*, and *pfDusp10*, were identified in the yellow catfish (*Pelteobagrus fulvidraco*) genome. Sequence and structural analysis revealed conserved domains, including a functional DSPc domain in all *pfDUSP* proteins. Phylogenetic analysis indicated that the evolution of the *pfDusp* genes aligns with species taxonomy. Expression profiles in the kidney following *A. hydrophila* infection showed that the *Dusp* gene family is involved in antibacterial immune responses. These findings suggest that *Dusp* genes are conserved and play a role in the immune response to bacterial infections in yellow catfish.

## Data Availability

The original contributions presented in the study are included in the article/**Supplementary Material**. Further inquiries can be directed to the corresponding author.
