# Supplementary material for: Genome-wide characterization and comparative expression profiling of dual-specificity phosphatase genes in yellow catfish (Pelteobagrus fulvidraco) after infection with exogenous Aeromonas hydrophila
Source: Front Immunol. 2024 Nov 13;15:1481696. doi: 10.3389/fimmu.2024.1481696 (PMC11598348; doi:10.3389/fimmu.2024.1481696)
Supplement: Supplementary file 1 [file Table1.docx]

Supplementary Material

**Table S1.** The amino acid sequences of the eight DUSP proteins in yellow catfish (*Pelteobagrus fulvidraco*).

DUSP1

| **1** | ATG | TAT | TTA | GAT | TTG | TCT | TAT | CCT | GTG | CGT | CGT | CCT | ACT | ATG | GTC | ATA | ATG | GAG | GTT | CCA | AAC | ATC | GAC | TGT | GGC | TCT | CTC | CGG | GCG | CTT | **90** |
| --- | --- | --- | --- | --- | --- | --- | --- | --- | --- | --- | --- | --- | --- | --- | --- | --- | --- | --- | --- | --- | --- | --- | --- | --- | --- | --- | --- | --- | --- | --- | --- |
| **1** | **M** | **Y** | **L** | **D** | **L** | **S** | **Y** | **P** | **V** | **R** | **R** | **P** | **T** | **M** | **V** | **I** | **M** | **E** | **V** | **P** | **N** | **I** | **D** | **C** | **G** | **S** | **L** | **R** | **A** | **L** | **30** |
| **91** | TTG | GAG | GGA | TCC | GAG | ACT | GGT | TGT | CTT | GTT | TTG | GAC | TGC | CGG | TCG | TTC | TTC | TCC | TTT | AGC | TCT | TCT | CAC | ATA | TCC | GGC | TCA | ACT | AAC | GTG | **180** |
| **31** | **L** | **E** | **G** | **S** | **E** | **T** | **G** | **C** | **L** | **V** | **L** | **D** | **C** | **R** | **S** | **F** | **F** | **S** | **F** | **S** | **S** | **S** | **H** | **I** | **S** | **G** | **S** | **T** | **N** | **V** | **60** |
| **181** | CGT | TTT | AGC | GCA | ATA | GTG | CGC | CGG | AGG | GCG | CGA | GGC | GGA | CTT | GGG | CTA | GAA | CAC | ATT | GTT | CCC | AAT | GAG | GAC | ACG | CGG | AGC | AAG | CTG | CTT | **270** |
| **61** | **R** | **F** | **S** | **A** | **I** | **V** | **R** | **R** | **R** | **A** | **R** | **G** | **G** | **L** | **G** | **L** | **E** | **H** | **I** | **V** | **P** | **N** | **E** | **D** | **T** | **R** | **S** | **K** | **L** | **L** | **90** |
| **271** | TCC | GGA | GAA | TAT | CAG | TCT | GTG | GTA | CTA | CTG | GAT | GAC | CGA | AGC | TTA | GAC | TTC | AGC | CAA | GTG | AAA | AAA | GAC | GGA | ACT | CTG | ATG | CTG | GCC | GTG | **360** |
| **91** | **S** | **G** | **E** | **Y** | **Q** | **S** | **V** | **V** | **L** | **L** | **D** | **D** | **R** | **S** | **L** | **D** | **F** | **S** | **Q** | **V** | **K** | **K** | **D** | **G** | **T** | **L** | **M** | **L** | **A** | **V** | **120** |
| **361** | ACC | GCG | TTG | AGT | CGG | AAC | AAC | CCC | CGA | GGA | GCG | CAC | GTC | TTC | TTC | TTG | AAA | GGC | GGC | TTC | GAC | ACC | TTT | TCA | TCC | GAG | TTC | CCT | GAA | ATG | **450** |
| **121** | **T** | **A** | **L** | **S** | **R** | **N** | **N** | **P** | **R** | **G** | **A** | **H** | **V** | **F** | **F** | **L** | **K** | **G** | **G** | **F** | **D** | **T** | **F** | **S** | **S** | **E** | **F** | **P** | **E** | **M** | **150** |
| **451** | TGT | ACC | AAA | GCA | CCA | CCT | CCA | CAA | GGA | CTG | AGT | TTG | CCT | CTC | AGT | GCC | AAC | TGT | CCT | CCT | GGT | AGC | GCC | GAA | CCA | AAC | TGT | AAT | AGC | TGT | **540** |
| **151** | **C** | **T** | **K** | **A** | **P** | **P** | **P** | **Q** | **G** | **L** | **S** | **L** | **P** | **L** | **S** | **A** | **N** | **C** | **P** | **P** | **G** | **S** | **A** | **E** | **P** | **N** | **C** | **N** | **S** | **C** | **180** |
| **541** | ACT | ACT | CCT | CTC | TAC | GAC | CAG | GGT | GGC | CCA | GTG | GAA | ATT | CTG | CCT | TTT | CTG | TAT | CTT | GGA | AGT | GCC | TAC | CAT | GCT | TCC | CGT | AAA | GAC | ATG | **630** |
| **181** | **T** | **T** | **P** | **L** | **Y** | **D** | **Q** | **G** | **G** | **P** | **V** | **E** | **I** | **L** | **P** | **F** | **L** | **Y** | **L** | **G** | **S** | **A** | **Y** | **H** | **A** | **S** | **R** | **K** | **D** | **M** | **210** |
| **631** | CTG | GAC | ATG | TTG | GGC | ATC | ACG | GCA | CTC | ATA | AAT | GTT | TCT | GCC | AAC | TGC | CCC | AAT | CAT | TTT | GAG | GAC | CAT | TAT | CAG | TAC | AAG | AGC | ATT | CCA | **720** |
| **211** | **L** | **D** | **M** | **L** | **G** | **I** | **T** | **A** | **L** | **I** | **N** | **V** | **S** | **A** | **N** | **C** | **P** | **N** | **H** | **F** | **E** | **D** | **H** | **Y** | **Q** | **Y** | **K** | **S** | **I** | **P** | **240** |
| **721** | GTT | GAA | GAC | AAC | CAC | AAG | GCA | GAC | ATT | AGC | TCC | TGG | TTC | AAT | GAG | GCC | ATC | GAA | TTT | ATC | GAC | TCT | GTG | AGA | AAC | AAA | GGT | GGC | CGT | GTC | **810** |
| **241** | **V** | **E** | **D** | **N** | **H** | **K** | **A** | **D** | **I** | **S** | **S** | **W** | **F** | **N** | **E** | **A** | **I** | **E** | **F** | **I** | **D** | **S** | **V** | **R** | **N** | **K** | **G** | **G** | **R** | **V** | **270** |
| **811** | TTT | GTG | CAC | TGC | CAG | GCG | GGC | ATC | TCG | CGC | TCG | GCA | ACC | ATC | TGT | CTG | GCC | TAC | CTC | ATG | CGT | ACC | AAC | CGT | GTC | AAG | TTG | GAC | GAG | GCT | **900** |
| **271** | **F** | **V** | **H** | **C** | **Q** | **A** | **G** | **I** | **S** | **R** | **S** | **A** | **T** | **I** | **C** | **L** | **A** | **Y** | **L** | **M** | **R** | **T** | **N** | **R** | **V** | **K** | **L** | **D** | **E** | **A** | **300** |
| **901** | TTC | GAG | TTC | GTC | AAG | CAG | CGC | CGC | AGT | ATC | ATC | TCA | CCC | AAC | TTC | AGC | TTC | ATG | GGC | CAG | CTT | CTG | CAG | TTC | GAG | TCA | CAA | GTG | CTT | GCT | **990** |
| **301** | **F** | **E** | **F** | **V** | **K** | **Q** | **R** | **R** | **S** | **I** | **I** | **S** | **P** | **N** | **F** | **S** | **F** | **M** | **G** | **Q** | **L** | **L** | **Q** | **F** | **E** | **S** | **Q** | **V** | **L** | **A** | **330** |
| **991** | ACG | TCC | ACC | TGC | TCA | TCA | GAG | GCT | AGC | AGT | CCA | GCA | CTC | AGC | AAA | AGT | GGC | ACT | GTT | TTC | AAC | TTT | CCT | GTC | TCT | ATT | CCT | GTC | CAT | GCT | **1080** |
| **331** | **T** | **S** | **T** | **C** | **S** | **S** | **E** | **A** | **S** | **S** | **P** | **A** | **L** | **S** | **K** | **S** | **G** | **T** | **V** | **F** | **N** | **F** | **P** | **V** | **S** | **I** | **P** | **V** | **H** | **A** | **360** |
| **1081** | GGT | GCC | AGT | CCA | CTG | TCC | TTC | TTG | TCC | CAT | CAT | AGT | CCC | ATA | ACT | CCT | TCA | CCT | ACT | TGC | TGA |  |  |  |  |  |  |  |  |  | **1170** |
| **361** | **G** | **A** | **S** | **P** | **L** | **S** | **F** | **L** | **S** | **H** | **H** | **S** | **P** | **I** | **T** | **P** | **S** | **P** | **T** | **C** | **.** |  |  |  |  |  |  |  |  |  | **390** |

DUSP2

| **1** | ATG | GGG | GTT | ATC | GGA | GAA | CCG | GTT | GAA | ATC | ACA | AGC | GAA | GAG | CTC | GTG | CAG | ATG | CTC | GTG | ACA | CCG | GAG | ATC | AGC | GGG | GCG | CTG | CTG | CTG | **90** |
| --- | --- | --- | --- | --- | --- | --- | --- | --- | --- | --- | --- | --- | --- | --- | --- | --- | --- | --- | --- | --- | --- | --- | --- | --- | --- | --- | --- | --- | --- | --- | --- |
| **1** | **M** | **G** | **V** | **I** | **G** | **E** | **P** | **V** | **E** | **I** | **T** | **S** | **E** | **E** | **L** | **V** | **Q** | **M** | **L** | **V** | **T** | **P** | **E** | **I** | **S** | **G** | **A** | **L** | **L** | **L** | **30** |
| **91** | CTG | GAC | TGC | CGT | CCG | TTC | CTT | GCT | TTC | TCG | CGC | GCG | CAC | ATC | TGC | CGC | GCG | CAC | AAC | GCC | GCG | TGG | AAC | TCG | CTG | CTG | CGG | CGC | AGG | TGC | **180** |
| **31** | **L** | **D** | **C** | **R** | **P** | **F** | **L** | **A** | **F** | **S** | **R** | **A** | **H** | **I** | **C** | **R** | **A** | **H** | **N** | **A** | **A** | **W** | **N** | **S** | **L** | **L** | **R** | **R** | **R** | **C** | **60** |
| **181** | AGC | CGT | GGC | ACG | GAA | TCC | GGC | GCG | CGC | CTC | GAC | TGC | CTG | CTC | GCC | GAC | AGG | TCT | CTG | CTG | GGG | CGC | GTG | CGC | ACA | GGG | GAG | TTC | CGC | CAG | **270** |
| **61** | **S** | **R** | **G** | **T** | **E** | **S** | **G** | **A** | **R** | **L** | **D** | **C** | **L** | **L** | **A** | **D** | **R** | **S** | **L** | **L** | **G** | **R** | **V** | **R** | **T** | **G** | **E** | **F** | **R** | **Q** | **90** |
| **271** | ATC | GTG | GTG | CTG | GAC | GAG | AAA | AGC | CGC | GCG | GTC | ACC | GAG | CTG | GAG | CGC | GAG | AGC | ATG | ACG | GGT | CTG | GTG | CTG | TGC | GCG | CTC | CAG | AGA | GAG | **360** |
| **91** | **I** | **V** | **V** | **L** | **D** | **E** | **K** | **S** | **R** | **A** | **V** | **T** | **E** | **L** | **E** | **R** | **E** | **S** | **M** | **T** | **G** | **L** | **V** | **L** | **C** | **A** | **L** | **Q** | **R** | **E** | **120** |
| **361** | CTG | CAC | GCG | GGC | ACG | GCG | CGG | ATC | TGC | TTC | CTG | CAA | GGT | GGA | TTC | GAT | GGT | TTC | TTG | GCG | CTC | TAC | CCT | GAG | CTG | TGT | ATC | AGT | GCA | CAG | **450** |
| **121** | **L** | **H** | **A** | **G** | **T** | **A** | **R** | **I** | **C** | **F** | **L** | **Q** | **G** | **G** | **F** | **D** | **G** | **F** | **L** | **A** | **L** | **Y** | **P** | **E** | **L** | **C** | **I** | **S** | **A** | **Q** | **150** |
| **451** | GAT | GTT | TGT | GGG | ACT | CAA | ACG | ACG | CTG | AGC | GAG | TCA | GAG | CAA | CGA | GTC | TCC | GGC | AGA | ACA | ACA | CCT | CTT | TAC | GAT | CAG | GGG | GGT | CCT | GTA | **540** |
| **151** | **D** | **V** | **C** | **G** | **T** | **Q** | **T** | **T** | **L** | **S** | **E** | **S** | **E** | **Q** | **R** | **V** | **S** | **G** | **R** | **T** | **T** | **P** | **L** | **Y** | **D** | **Q** | **G** | **G** | **P** | **V** | **180** |
| **541** | GAG | ATC | TTG | CCC | TTT | CTG | TTT | CTG | GGC | AGC | GCT | CAT | CAT | TCG | TCT | CAG | AGG | GAG | CTG | CTG | AGG | CGC | TGC | AAC | ATC | ACA | GCC | GTC | CTT | AAC | **630** |
| **181** | **E** | **I** | **L** | **P** | **F** | **L** | **F** | **L** | **G** | **S** | **A** | **H** | **H** | **S** | **S** | **Q** | **R** | **E** | **L** | **L** | **R** | **R** | **C** | **N** | **I** | **T** | **A** | **V** | **L** | **N** | **210** |
| **631** | GTC | TCC | TCG | TCC | TGC | CCC | AAT | CTG | TTT | GAG | CAT | GAG | CTG | AGC | TAC | ATG | ACC | CTG | AGG | GTG | GAG | GAC | AGC | ATG | GCG | GCG | GAC | ATC | CGA | GTG | **720** |
| **211** | **V** | **S** | **S** | **S** | **C** | **P** | **N** | **L** | **F** | **E** | **H** | **E** | **L** | **S** | **Y** | **M** | **T** | **L** | **R** | **V** | **E** | **D** | **S** | **M** | **A** | **A** | **D** | **I** | **R** | **V** | **240** |
| **721** | CTC | TTC | CCA | AAG | GCC | ATC | CAC | TTT | ATC | GAT | TCA | GTG | AAA | GAA | AGT | GGC | GGT | CGA | GTT | CTG | GTC | CAC | TGC | CAG | GCC | GGC | ATC | TCC | CGC | TCG | **810** |
| **241** | **L** | **F** | **P** | **K** | **A** | **I** | **H** | **F** | **I** | **D** | **S** | **V** | **K** | **E** | **S** | **G** | **G** | **R** | **V** | **L** | **V** | **H** | **C** | **Q** | **A** | **G** | **I** | **S** | **R** | **S** | **270** |
| **811** | GCC | ACA | ATC | TGC | CTG | GCG | TAT | CTA | ATC | CAC | GCC | CGC | CGC | GTG | CGC | CTG | AAC | GAG | GCT | TTC | GAG | TTC | GTG | AAG | CGC | CGA | CGA | CAG | GTC | ATC | **900** |
| **271** | **A** | **T** | **I** | **C** | **L** | **A** | **Y** | **L** | **I** | **H** | **A** | **R** | **R** | **V** | **R** | **L** | **N** | **E** | **A** | **F** | **E** | **F** | **V** | **K** | **R** | **R** | **R** | **Q** | **V** | **I** | **300** |
| **901** | TCC | CCA | AAC | CTG | GCC | TTC | ATG | GGC | CAG | CTA | CTG | CAG | TTC | GAG | ACA | GAC | GTT | CTG | TGT | CCT | TAC | ACG | GTT | CTG | GAC | ACG | GAG | GAT | GGC | GCC | **990** |
| **301** | **S** | **P** | **N** | **L** | **A** | **F** | **M** | **G** | **Q** | **L** | **L** | **Q** | **F** | **E** | **T** | **D** | **V** | **L** | **C** | **P** | **Y** | **T** | **V** | **L** | **D** | **T** | **E** | **D** | **G** | **A** | **330** |
| **991** | ACT | GCA | TTT | CAG | TAC | CTC | TGC | ACA | GAC | AAC | ACA | GAC | CTT | TCA | TGA |  |  |  |  |  |  |  |  |  |  |  |  |  |  |  | **1080** |
| **331** | **T** | **A** | **F** | **Q** | **Y** | **L** | **C** | **T** | **D** | **N** | **T** | **D** | **L** | **S** | **.** |  |  |  |  |  |  |  |  |  |  |  |  |  |  |  | **360** |

DUSP3

| **1** | ATG | ACA | CTT | GTT | TGG | ACT | GAA | AGG | CGT | CTT | GAA | ACC | ATG | AAG | AAG | CAG | CAC | CAA | AGC | CCC | GCT | AAG | CTC | GCG | GCA | GAA | GTG | CGC | GTG | CCA | **90** |  |
| --- | --- | --- | --- | --- | --- | --- | --- | --- | --- | --- | --- | --- | --- | --- | --- | --- | --- | --- | --- | --- | --- | --- | --- | --- | --- | --- | --- | --- | --- | --- | --- | --- |
| **1** | **M** | **T** | **L** | **V** | **W** | **T** | **E** | **R** | **R** | **L** | **E** | **T** | **M** | **K** | **K** | **Q** | **H** | **Q** | **S** | **P** | **A** | **K** | **L** | **A** | **A** | **E** | **V** | **R** | **V** | **P** | **30** |  |
| **91** | GAA | GGC | GAA | GCT | ACC | GTG | CAG | CAA | CTT | AAC | GAG | CTT | TTG | TCT | GAC | AGC | AGC | GGC | TTT | TAC | ACT | TTG | CCA | GCG | CAA | CAT | TTC | AAC | GAG | GTG | **180** |  |
| **31** | **E** | **G** | **E** | **A** | **T** | **V** | **Q** | **Q** | **L** | **N** | **E** | **L** | **L** | **S** | **D** | **S** | **S** | **G** | **F** | **Y** | **T** | **L** | **P** | **A** | **Q** | **H** | **F** | **N** | **E** | **V** | **60** |  |
| **181** | TTT | CCT | AGG | ATT | TAC | ATC | GGG | AAC | GCA | TTT | GTG | GCC | CAG | AAT | GTG | ATG | CGT | CTG | CAG | CGG | CTC | GGC | ATC | ACA | CAC | ATA | CTC | AAC | ACA | GCG | **270** |  |
| **61** | **F** | **P** | **R** | **I** | **Y** | **I** | **G** | **N** | **A** | **F** | **V** | **A** | **Q** | **N** | **V** | **M** | **R** | **L** | **Q** | **R** | **L** | **G** | **I** | **T** | **H** | **I** | **L** | **N** | **T** | **A** | **90** |  |
| **271** | GAG | GGC | AAC | TCC | TTC | ATG | CAC | GTG | AAC | ACC | AAC | GCT | GAG | TTC | TAT | GCA | GGA | AGT | GGG | ATC | ACA | TAC | CAT | GGC | ATA | AAG | GCC | AAT | GAC | ACG | **360** |  |
| **91** | **E** | **G** | **N** | **S** | **F** | **M** | **H** | **V** | **N** | **T** | **N** | **A** | **E** | **F** | **Y** | **A** | **G** | **S** | **G** | **I** | **T** | **Y** | **H** | **G** | **I** | **K** | **A** | **N** | **D** | **T** | **120** |  |
| **361** | GAA | CAG | TTT | AAC | CTC | TGT | GCC | TTC | TTT | GAG | GAA | GGG | GCC | GAC | TTC | ATT | GAT | AAG | GCT | CTG | GCA | CAT | GCA | AAT | GGA | AAA | GGA | AAA | GTG | TAC | **450** |  |
| **121** | **E** | **Q** | **F** | **N** | **L** | **C** | **A** | **F** | **F** | **E** | **E** | **G** | **A** | **D** | **F** | **I** | **D** | **K** | **A** | **L** | **A** | **H** | **A** | **N** | **G** | **K** | **G** | **K** | **V** | **Y** | **150** |  |
| **451** | GTT | CAC | TGC | CGA | GAA | GGC | TAC | AGC | CGC | TCC | CCG | ACT | ATT | GTC | ATT | GCT | TAC | CTC | ATG | CTG | CGC | CAT | AAA | ATG | GAT | GTG | CGA | GTG | GCG | ACG | **540** |  |
| **151** | **V** | **H** | **C** | **R** | **E** | **G** | **Y** | **S** | **R** | **S** | **P** | **T** | **I** | **V** | **I** | **A** | **Y** | **L** | **M** | **L** | **R** | **H** | **K** | **M** | **D** | **V** | **R** | **V** | **A** | **T** | **180** |  |
| **541** | GCT | ACT | GTA | AGG | CAC | AAG | AGA | GAA | ATC | GGG | CCC | AAC | GAT | GGC | TTC | CTG | CGC | CAG | CTG | TGC | CAA | CTC | AAC | GAG | AAG | CTG | GCC | AAA | GAG | GGC | **630** |  |
| **181** | **A** | **T** | **V** | **R** | **H** | **K** | **R** | **E** | **I** | **G** | **P** | **N** | **D** | **G** | **F** | **L** | **R** | **Q** | **L** | **C** | **Q** | **L** | **N** | **E** | **K** | **L** | **A** | **K** | **E** | **G** | **210** |  |
| **631** | AAG | CTG | AAG | ACT | AAA | TGA |  |  |  |  |  |  |  |  |  |  |  |  |  |  |  |  |  |  |  |  |  |  |  |  | **720** |  |
| **211** | **K** | **L** | **K** | **T** | **K** | **.** |  |  |  |  |  |  |  |  |  |  |  |  |  |  |  |  |  |  |  |  |  |  |  |  | **240** |  |

DUSP4

| **1** | ATG | GAA | CAG | CTG | AGC | GAG | ATG | GAG | TGC | GGA | GTT | CTG | AAG | CGG | CTG | CTG | AAG | GAG | GAC | GGA | ACG | GCG | CGG | TGC | CTG | GTG | TTG | GAC | TGT | CGC | **90** |
| --- | --- | --- | --- | --- | --- | --- | --- | --- | --- | --- | --- | --- | --- | --- | --- | --- | --- | --- | --- | --- | --- | --- | --- | --- | --- | --- | --- | --- | --- | --- | --- |
| **1** | **M** | **E** | **Q** | **L** | **S** | **E** | **M** | **E** | **C** | **G** | **V** | **L** | **K** | **R** | **L** | **L** | **K** | **E** | **D** | **G** | **T** | **A** | **R** | **C** | **L** | **V** | **L** | **D** | **C** | **R** | **30** |
| **91** | TCC | TTC | CTG | GCG | TTC | AGC | GCC | TGC | CAC | ATA | CGG | AGC | GCG | GTG | AAC | GTC | CGC | TGC | AAC | ACG | ATA | GTG | CGC | CGG | AGA | GCG | AAG | GGC | TCC | GTG | **180** |
| **31** | **S** | **F** | **L** | **A** | **F** | **S** | **A** | **C** | **H** | **I** | **R** | **S** | **A** | **V** | **N** | **V** | **R** | **C** | **N** | **T** | **I** | **V** | **R** | **R** | **R** | **A** | **K** | **G** | **S** | **V** | **60** |
| **181** | TCT | CTG | GAC | CAG | ATC | CTG | GCT | GGG | GAT | GAA | GAG | GCG | AAG | TGC | AGA | CTG | ATC | TCA | GGC | CTG | TAT | TCT | GCC | GTC | ATC | CTT | TAC | GAC | GAG | CGC | **270** |
| **61** | **S** | **L** | **D** | **Q** | **I** | **L** | **A** | **G** | **D** | **E** | **E** | **A** | **K** | **C** | **R** | **L** | **I** | **S** | **G** | **L** | **Y** | **S** | **A** | **V** | **I** | **L** | **Y** | **D** | **E** | **R** | **90** |
| **271** | ACA | CCG | GAT | GCG | GCC | ACT | CTC | AAA | GAG | GAC | AGC | ACC | ATC | GCG | CTG | GTG | TTT | AAC | GCA | CTG | GGC | AGG | GAC | ACC | TTT | AAC | ACA | GAG | GTC | TAT | **360** |
| **91** | **T** | **P** | **D** | **A** | **A** | **T** | **L** | **K** | **E** | **D** | **S** | **T** | **I** | **A** | **L** | **V** | **F** | **N** | **A** | **L** | **G** | **R** | **D** | **T** | **F** | **N** | **T** | **E** | **V** | **Y** | **120** |
| **361** | CTC | CTC | AAA | GGA | GGC | TAC | GAC | AGA | TTC | TTC | TCT | CTA | TAT | CCA | GAC | TAC | TGT | TTA | AAG | AGT | AAA | TCT | CTG | CCG | ACG | CTC | GCA | GCT | CAG | AGC | **450** |
| **121** | **L** | **L** | **K** | **G** | **G** | **Y** | **D** | **R** | **F** | **F** | **S** | **L** | **Y** | **P** | **D** | **Y** | **C** | **L** | **K** | **S** | **K** | **S** | **L** | **P** | **T** | **L** | **A** | **A** | **Q** | **S** | **150** |
| **451** | AGC | ACT | GAG | ACG | AGC | TGC | ATC | TCC | TGC | ACA | ACT | CCA | CAA | CAC | GAC | CAG | GGT | GGA | CCA | GTG | GAG | ATC | CTT | CCC | TTC | CTG | TTT | CTC | GGC | AGT | **540** |
| **151** | **S** | **T** | **E** | **T** | **S** | **C** | **I** | **S** | **C** | **T** | **T** | **P** | **Q** | **H** | **D** | **Q** | **G** | **G** | **P** | **V** | **E** | **I** | **L** | **P** | **F** | **L** | **F** | **L** | **G** | **S** | **180** |
| **541** | GCC | TTT | CAT | GCA | TCC | AAA | AAA | GAC | ATG | TTG | GAT | GGC | ATG | GGC | ATC | TCT | GCC | CTG | CTG | AAT | GTG | TCT | TCA | AAT | TGT | CCC | AAC | CAC | TTT | GAA | **630** |
| **181** | **A** | **F** | **H** | **A** | **S** | **K** | **K** | **D** | **M** | **L** | **D** | **G** | **M** | **G** | **I** | **S** | **A** | **L** | **L** | **N** | **V** | **S** | **S** | **N** | **C** | **P** | **N** | **H** | **F** | **E** | **210** |
| **631** | GGC | GTT | TAC | CAG | TAT | AAG | TGC | ATC | CCA | GTG | GAG | GAC | AAC | CAC | AAG | GAG | GAC | ATC | AGC | TCC | TGG | TTC | ACC | GAA | GCC | ATT | GAC | TTC | ATA | GAC | **720** |
| **211** | **G** | **V** | **Y** | **Q** | **Y** | **K** | **C** | **I** | **P** | **V** | **E** | **D** | **N** | **H** | **K** | **E** | **D** | **I** | **S** | **S** | **W** | **F** | **T** | **E** | **A** | **I** | **D** | **F** | **I** | **D** | **240** |
| **721** | TCT | GTG | AAA | GAC | TCC | AAC | GGT | CGT | GTC | CTT | GTT | CAC | TGT | CAA | GCA | GGT | ATT | TCA | CGG | TCA | GCC | ACA | ATT | TGT | CTG | GCA | TAC | CTG | ATG | AAG | **810** |
| **241** | **S** | **V** | **K** | **D** | **S** | **N** | **G** | **R** | **V** | **L** | **V** | **H** | **C** | **Q** | **A** | **G** | **I** | **S** | **R** | **S** | **A** | **T** | **I** | **C** | **L** | **A** | **Y** | **L** | **M** | **K** | **270** |
| **811** | AAG | AAA | CGT | GTG | TGC | CTG | GAT | GAG | GCT | TTT | GAG | TTT | GTC | AAG | CAG | CGG | CGC | AGC | ATC | ATC | TCA | CCC | AAC | TTC | AGC | TTC | ATG | GGT | CAG | CTG | **900** |
| **271** | **K** | **K** | **R** | **V** | **C** | **L** | **D** | **E** | **A** | **F** | **E** | **F** | **V** | **K** | **Q** | **R** | **R** | **S** | **I** | **I** | **S** | **P** | **N** | **F** | **S** | **F** | **M** | **G** | **Q** | **L** | **300** |
| **901** | CTA | CAG | TTT | GAG | TCA | CAG | GTG | CTG | GCC | ACA | TCA | TGC | TCA | GTG | GAG | GCA | GCC | AGC | CCA | TCA | GCC | ACT | CTA | GGC | CCC | AAG | TCT | TCC | CCA | TCA | **990** |
| **301** | **L** | **Q** | **F** | **E** | **S** | **Q** | **V** | **L** | **A** | **T** | **S** | **C** | **S** | **V** | **E** | **A** | **A** | **S** | **P** | **S** | **A** | **T** | **L** | **G** | **P** | **K** | **S** | **S** | **P** | **S** | **330** |
| **991** | ACA | AGC | TCG | CCC | TTT | ATC | TTC | AGC | TTC | CCT | ATG | GGG | CCA | CAC | GGG | CAG | CCC | AGT | AGC | CTC | TCA | TAC | TTG | CAG | AGC | CCT | ATT | ACC | ACC | TCA | **1080** |
| **331** | **T** | **S** | **S** | **P** | **F** | **I** | **F** | **S** | **F** | **P** | **M** | **G** | **P** | **H** | **G** | **Q** | **P** | **S** | **S** | **L** | **S** | **Y** | **L** | **Q** | **S** | **P** | **I** | **T** | **T** | **S** | **360** |
| **1081** | CCT | AGC | TGC | TGA |  |  |  |  |  |  |  |  |  |  |  |  |  |  |  |  |  |  |  |  |  |  |  |  |  |  | **1170** |
| **361** | **P** | **S** | **C** | **.** |  |  |  |  |  |  |  |  |  |  |  |  |  |  |  |  |  |  |  |  |  |  |  |  |  |  | **390** |

DUSP5

| **1** | ATG | ATC | ATG | AAG | GTT | TCC | AGT | ATA | GAC | AGC | CGT | CAT | TTC | AAG | AAA | ATC | CTG | CGC | AAG | GAG | ACC | GGG | AAG | TGT | TTG | ATC | CTG | GAC | TGT | AGA | **90** |
| --- | --- | --- | --- | --- | --- | --- | --- | --- | --- | --- | --- | --- | --- | --- | --- | --- | --- | --- | --- | --- | --- | --- | --- | --- | --- | --- | --- | --- | --- | --- | --- |
| **1** | **M** | **I** | **M** | **K** | **V** | **S** | **S** | **I** | **D** | **S** | **R** | **H** | **F** | **K** | **K** | **I** | **L** | **R** | **K** | **E** | **T** | **G** | **K** | **C** | **L** | **I** | **L** | **D** | **C** | **R** | **30** |
| **91** | CCG | TAT | TTA | TCG | TTC | TCC | AGC | TCG | AGC | GTC | CGC | GGC | TCG | GTG | AAC | GTT | AAC | CTG | AAC | TCG | GTG | GTG | CTG | CGC | AGG | TCG | CGC | GGG | GCT | CCG | **180** |
| **31** | **P** | **Y** | **L** | **S** | **F** | **S** | **S** | **S** | **S** | **V** | **R** | **G** | **S** | **V** | **N** | **V** | **N** | **L** | **N** | **S** | **V** | **V** | **L** | **R** | **R** | **S** | **R** | **G** | **A** | **P** | **60** |
| **181** | GTG | CCG | CTG | CGG | TTC | GTT | GTG | CCG | GAT | GAG | CAC | GCG | CTG | TAC | CGG | CTG | CGC | GAG | GGC | GGC | GTG | TCG | GTG | GTG | GTG | GCG | CTG | GAC | GAG | AAC | **270** |
| **61** | **V** | **P** | **L** | **R** | **F** | **V** | **V** | **P** | **D** | **E** | **H** | **A** | **L** | **Y** | **R** | **L** | **R** | **E** | **G** | **G** | **V** | **S** | **V** | **V** | **V** | **A** | **L** | **D** | **E** | **N** | **90** |
| **271** | ACG | CCG | CAT | CTC | CAC | AAG | CTG | AAG | AAG | GAC | AGC | GTG | GCG | CGT | CTC | GCC | ATC | CAC | AGC | CTC | GCG | CAC | CTC | TCC | AGC | TGT | GCC | AAC | ATC | TGC | **360** |
| **91** | **T** | **P** | **H** | **L** | **H** | **K** | **L** | **K** | **K** | **D** | **S** | **V** | **A** | **R** | **L** | **A** | **I** | **H** | **S** | **L** | **A** | **H** | **L** | **S** | **S** | **C** | **A** | **N** | **I** | **C** | **120** |
| **361** | TTC | CTG | AAG | GGA | GGC | TAC | GAG | AGC | TTC | CAG | GCG | CAT | TAC | CCC | GAG | TTG | TGT | ACC | GAG | GCC | AAA | GCA | CCT | CTG | GAG | CGC | AGT | GTG | CCG | GAC | **450** |
| **121** | **F** | **L** | **K** | **G** | **G** | **Y** | **E** | **S** | **F** | **Q** | **A** | **H** | **Y** | **P** | **E** | **L** | **C** | **T** | **E** | **A** | **K** | **A** | **P** | **L** | **E** | **R** | **S** | **V** | **P** | **D** | **150** |
| **451** | ACA | GAG | GTT | TAT | CCT | GGG | ACA | GAT | TAC | ACT | CAG | GAT | GGT | CCG | GTG | GAG | CTG | TTG | CCC | TTC | CTG | TAC | CTG | GGC | AGT | GCT | CAC | CAT | GCA | CGC | **540** |
| **151** | **T** | **E** | **V** | **Y** | **P** | **G** | **T** | **D** | **Y** | **T** | **Q** | **D** | **G** | **P** | **V** | **E** | **L** | **L** | **P** | **F** | **L** | **Y** | **L** | **G** | **S** | **A** | **H** | **H** | **A** | **R** | **180** |
| **541** | AGA | CAC | GAC | TGC | CTG | AGT | GAG | CTG | CGC | ATC | ACG | GCG | CTG | TTG | AAT | GTT | TCA | CGG | CGA | GAC | TGG | CAG | TGT | GCC | GGA | GGG | CCA | CAG | CGG | TAC | **630** |
| **181** | **R** | **H** | **D** | **C** | **L** | **S** | **E** | **L** | **R** | **I** | **T** | **A** | **L** | **L** | **N** | **V** | **S** | **R** | **R** | **D** | **W** | **Q** | **C** | **A** | **G** | **G** | **P** | **Q** | **R** | **Y** | **210** |
| **631** | AAA | CGC | ATC | GCT | GTA | GAG | GAC | AGT | CAC | ACA | GCT | GAT | ATT | GGC | TCA | CAC | TTC | CAG | GAA | GCT | ATT | GAC | TTC | ATT | GAC | GAG | GTG | AAG | CGA | GAA | **720** |
| **211** | **K** | **R** | **I** | **A** | **V** | **E** | **D** | **S** | **H** | **T** | **A** | **D** | **I** | **G** | **S** | **H** | **F** | **Q** | **E** | **A** | **I** | **D** | **F** | **I** | **D** | **E** | **V** | **K** | **R** | **E** | **240** |
| **721** | GGC | GGG | AAG | GTG | CTG | GTG | CAC | TGC | GAG | GCA | GGG | ATT | TCA | CGC | TCG | CCC | ACC | ATC | TGT | ATG | GCG | TAC | CTC | ATG | AAG | ACA | CAG | AGG | CTG | CGT | **810** |
| **241** | **G** | **G** | **K** | **V** | **L** | **V** | **H** | **C** | **E** | **A** | **G** | **I** | **S** | **R** | **S** | **P** | **T** | **I** | **C** | **M** | **A** | **Y** | **L** | **M** | **K** | **T** | **Q** | **R** | **L** | **R** | **270** |
| **811** | CTG | GAG | GAG | GCT | TTC | GAT | GCC | GTG | AGA | CAG | CGG | CGT | GCC | GTC | ATC | TCG | CCC | AAC | TTC | AGC | TTC | ATG | GGT | CAG | CTG | CTG | CAG | TTC | GAG | AAC | **900** |
| **271** | **L** | **E** | **E** | **A** | **F** | **D** | **A** | **V** | **R** | **Q** | **R** | **R** | **A** | **V** | **I** | **S** | **P** | **N** | **F** | **S** | **F** | **M** | **G** | **Q** | **L** | **L** | **Q** | **F** | **E** | **N** | **300** |
| **901** | GAG | GTC | CTG | GCG | TCT | GTG | CCC | GAC | AGC | AAT | ATG | GAA | AAC | AGC | AAT | GAC | CAA | CAA | AAG | AGT | GAC | GAG | TTC | ACG | ATC | GAC | AAG | AGC | TTC | GAG | **990** |
| **301** | **E** | **V** | **L** | **A** | **S** | **V** | **P** | **D** | **S** | **N** | **M** | **E** | **N** | **S** | **N** | **D** | **Q** | **Q** | **K** | **S** | **D** | **E** | **F** | **T** | **I** | **D** | **K** | **S** | **F** | **E** | **330** |
| **991** | TCA | TCC | GTT | TTC | TCC | TTC | CCT | ACC | TCC | TTC | CTG | TCA | CCC | ATC | AAA | CTC | AAT | CCC | ATC | ACC | TCC | CTG | ACT | ACT | TGA |  |  |  |  |  | **1080** |
| **331** | **S** | **S** | **V** | **F** | **S** | **F** | **P** | **T** | **S** | **F** | **L** | **S** | **P** | **I** | **K** | **L** | **N** | **P** | **I** | **T** | **S** | **L** | **T** | **T** | **.** |  |  |  |  |  | **360** |

DUSP6

| **1** | ATG | CTC | GAT | AAG | TTC | CGG | CCC | GCG | CAG | CTC | GAC | ACG | GTA | ATG | GCG | ATC | AGC | AAG | AGC | GTG | GCG | TGG | CTC | AGG | GAG | CAG | TTG | GAG | ACG | CGC | **90** |
| --- | --- | --- | --- | --- | --- | --- | --- | --- | --- | --- | --- | --- | --- | --- | --- | --- | --- | --- | --- | --- | --- | --- | --- | --- | --- | --- | --- | --- | --- | --- | --- |
| **1** | **M** | **L** | **D** | **K** | **F** | **R** | **P** | **A** | **Q** | **L** | **D** | **T** | **V** | **M** | **A** | **I** | **S** | **K** | **S** | **V** | **A** | **W** | **L** | **R** | **E** | **Q** | **L** | **E** | **T** | **R** | **30** |
| **91** | CGC | GAG | CGC | CTG | CTC | GTG | ATG | GAC | TGC | CGC | GCG | CGA | GAG | CTC | TAC | GAC | TCG | TCG | CAC | GTC | GAG | GCG | GCC | ATC | AAC | GTG | GCC | ATC | CCG | AGT | **180** |
| **31** | **R** | **E** | **R** | **L** | **L** | **V** | **M** | **D** | **C** | **R** | **A** | **R** | **E** | **L** | **Y** | **D** | **S** | **S** | **H** | **V** | **E** | **A** | **A** | **I** | **N** | **V** | **A** | **I** | **P** | **S** | **60** |
| **181** | CTC | ATG | CTG | CGG | CGA | CTC | AAG | AAG | GGC | AAC | CTG | CCC | GTC | AAG | TCG | CTG | CTG | TCC | GAC | GGA | CAG | GAT | CGC | GAG | AGG | TTC | GCG | CGG | CGC | TGC | **270** |
| **61** | **L** | **M** | **L** | **R** | **R** | **L** | **K** | **K** | **G** | **N** | **L** | **P** | **V** | **K** | **S** | **L** | **L** | **S** | **D** | **G** | **Q** | **D** | **R** | **E** | **R** | **F** | **A** | **R** | **R** | **C** | **90** |
| **271** | AGG | ACC | GAC | ACT | ATC | GTG | CTG | TAC | GAC | GAG | TGC | AGT | CGC | GAG | TGG | AAC | GAG | AAC | GTG | GAC | GGC | GGC | TCG | GTG | CTC | GGT | TTA | CTG | CTA | CGG | **360** |
| **91** | **R** | **T** | **D** | **T** | **I** | **V** | **L** | **Y** | **D** | **E** | **C** | **S** | **R** | **E** | **W** | **N** | **E** | **N** | **V** | **D** | **G** | **G** | **S** | **V** | **L** | **G** | **L** | **L** | **L** | **R** | **120** |
| **361** | AGG | ATG | AAG | GAC | GAG | GGA | TAC | AAA | GCC | TTT | TAC | CTT | GAA | GGC | GGT | TTC | AGC | AAG | TTT | CAG | TCC | GAG | TGT | CCT | GCG | CTC | TGC | GAG | ACC | AAC | **450** |
| **121** | **R** | **M** | **K** | **D** | **E** | **G** | **Y** | **K** | **A** | **F** | **Y** | **L** | **E** | **G** | **G** | **F** | **S** | **K** | **F** | **Q** | **S** | **E** | **C** | **P** | **A** | **L** | **C** | **E** | **T** | **N** | **150** |
| **451** | CTG | GAC | GGT | TCG | TGC | TGT | GGA | AGC | GGC | TCT | CCC | ACA | TCA | CAT | GTT | CTG | GGG | CTC | GGT | GGC | CTG | CGC | ATC | AGC | TCT | GAT | TCA | TCG | GAC | ATC | **540** |
| **151** | **L** | **D** | **G** | **S** | **C** | **C** | **G** | **S** | **G** | **S** | **P** | **T** | **S** | **H** | **V** | **L** | **G** | **L** | **G** | **G** | **L** | **R** | **I** | **S** | **S** | **D** | **S** | **S** | **D** | **I** | **180** |
| **541** | GAG | TCG | GAC | GCG | GAC | CGT | GAA | CCC | GGC | AGC | GCC | ACC | GAC | TCA | GAC | GGC | AGC | CCG | GCG | TCA | AAC | CCG | CAG | CCG | TCA | TTC | CCA | GTC | GAG | ATC | **630** |
| **181** | **E** | **S** | **D** | **A** | **D** | **R** | **E** | **P** | **G** | **S** | **A** | **T** | **D** | **S** | **D** | **G** | **S** | **P** | **A** | **S** | **N** | **P** | **Q** | **P** | **S** | **F** | **P** | **V** | **E** | **I** | **210** |
| **631** | CTG | CCA | CAT | CTG | TAC | CTA | GGC | TGT | GCA | AAG | GAC | TCC | ACC | AAC | CTG | GAC | GTG | CTG | GAG | GAG | TTC | GGC | ATC | AAG | TAC | ATC | CTG | AAC | GTG | ACC | **720** |
| **211** | **L** | **P** | **H** | **L** | **Y** | **L** | **G** | **C** | **A** | **K** | **D** | **S** | **T** | **N** | **L** | **D** | **V** | **L** | **E** | **E** | **F** | **G** | **I** | **K** | **Y** | **I** | **L** | **N** | **V** | **T** | **240** |
| **721** | CCG | AAC | CTG | CCC | AAC | CTT | TTC | GAA | AAC | GCA | GGA | GAA | TTC | AAG | TAC | AAA | CAG | ATT | CCT | ATA | TCG | GAC | CAC | TGG | AGC | CAG | AAC | CTC | TCG | CAG | **810** |
| **241** | **P** | **N** | **L** | **P** | **N** | **L** | **F** | **E** | **N** | **A** | **G** | **E** | **F** | **K** | **Y** | **K** | **Q** | **I** | **P** | **I** | **S** | **D** | **H** | **W** | **S** | **Q** | **N** | **L** | **S** | **Q** | **270** |
| **811** | TTC | TTC | CCA | GAG | GCC | ATC | GGC | TTC | ATC | GAC | GAG | GCT | CGA | GGT | CAG | AAG | TGT | GGC | GTC | CTC | GTG | CAC | TGT | CTC | GCT | GGT | ATT | AGC | CGC | TCC | **900** |
| **271** | **F** | **F** | **P** | **E** | **A** | **I** | **G** | **F** | **I** | **D** | **E** | **A** | **R** | **G** | **Q** | **K** | **C** | **G** | **V** | **L** | **V** | **H** | **C** | **L** | **A** | **G** | **I** | **S** | **R** | **S** | **300** |
| **901** | GTC | ACG | GTG | ACC | GTG | GCC | TAT | CTG | ATG | CAG | AAG | CTC | AAC | CTG | TCC | ATG | AAT | GAT | GCC | TAC | GAC | ATT | GTC | AAG | ATG | AAA | AAA | TCG | AAC | ATC | **990** |
| **301** | **V** | **T** | **V** | **T** | **V** | **A** | **Y** | **L** | **M** | **Q** | **K** | **L** | **N** | **L** | **S** | **M** | **N** | **D** | **A** | **Y** | **D** | **I** | **V** | **K** | **M** | **K** | **K** | **S** | **N** | **I** | **330** |
| **991** | TCG | CCC | AAC | TTT | AAC | TTC | ATG | GGG | CAG | CTG | TTG | GAC | TTT | GAG | CGC | ACG | CTC | GGG | CTG | CAG | AGC | CCA | TGC | GAC | AAC | CGA | GCT | TCG | GCG | CCT | **1080** |
| **331** | **S** | **P** | **N** | **F** | **N** | **F** | **M** | **G** | **Q** | **L** | **L** | **D** | **F** | **E** | **R** | **T** | **L** | **G** | **L** | **Q** | **S** | **P** | **C** | **D** | **N** | **R** | **A** | **S** | **A** | **P** | **360** |
| **1081** | TCG | CAG | CCG | CTC | TAC | TTC | AGC | ACG | CCC | ACC | AAT | CAC | AAT | GTC | TTC | CAG | CTC | GAC | CCG | CTC | GAG | TCC | ACG | TGA |  |  |  |  |  |  | **1170** |
| **361** | **S** | **Q** | **P** | **L** | **Y** | **F** | **S** | **T** | **P** | **T** | **N** | **H** | **N** | **V** | **F** | **Q** | **L** | **D** | **P** | **L** | **E** | **S** | **T** | **.** |  |  |  |  |  |  | **390** |

DUSP7

| **1** | ATG | TGG | GGG | AAA | AGT | GCG | GCA | TGG | CTG | CAG | GAC | GAG | CTG | GAG | TCT | GGG | CTC | AAC | TCT | CTG | CTC | CTG | CTC | GAC | TGT | CGC | TCT | CAC | GAG | CTT | **90** |
| --- | --- | --- | --- | --- | --- | --- | --- | --- | --- | --- | --- | --- | --- | --- | --- | --- | --- | --- | --- | --- | --- | --- | --- | --- | --- | --- | --- | --- | --- | --- | --- |
| **1** | **M** | **W** | **G** | **K** | **S** | **A** | **A** | **W** | **L** | **Q** | **D** | **E** | **L** | **E** | **S** | **G** | **L** | **N** | **S** | **L** | **L** | **L** | **L** | **D** | **C** | **R** | **S** | **H** | **E** | **L** | **30** |
| **91** | TAC | GAG | TCC | TCA | CAC | ATC | GAG | TCC | GCC | ATC | CAT | CTC | GCC | ATC | CCG | GGC | CTC | ATG | CTC | CGC | CGC | CTG | CGC | AAA | GGC | AAC | CTG | CCG | ATC | CGC | **180** |
| **31** | **Y** | **E** | **S** | **S** | **H** | **I** | **E** | **S** | **A** | **I** | **H** | **L** | **A** | **I** | **P** | **G** | **L** | **M** | **L** | **R** | **R** | **L** | **R** | **K** | **G** | **N** | **L** | **P** | **I** | **R** | **60** |
| **181** | TCC | GTC | ATC | CCC | AAC | GAC | ACG | GAC | AAG | GAG | CGC | TTC | ATC | CGC | CGC | TGC | AAG | AGC | GAC | ACC | GTG | CTG | CTG | TAT | GAC | GAG | AGC | GGG | GAC | AGC | **270** |
| **61** | **S** | **V** | **I** | **P** | **N** | **D** | **T** | **D** | **K** | **E** | **R** | **F** | **I** | **R** | **R** | **C** | **K** | **S** | **D** | **T** | **V** | **L** | **L** | **Y** | **D** | **E** | **S** | **G** | **D** | **S** | **90** |
| **271** | GGG | GCG | GCT | GCT | GCG | GGC | AGT | GCG | GGG | TCC | GTG | CTG | GGG | CTC | CTC | ATG | CAC | AGA | CTC | CGG | GAA | GAC | GGC | TGT | AAG | GCC | TAC | TAC | CTG | GAA | **360** |
| **91** | **G** | **A** | **A** | **A** | **A** | **G** | **S** | **A** | **G** | **S** | **V** | **L** | **G** | **L** | **L** | **M** | **H** | **R** | **L** | **R** | **E** | **D** | **G** | **C** | **K** | **A** | **Y** | **Y** | **L** | **E** | **120** |
| **361** | GGA | GGC | TTT | AAT | AAG | TTC | CAG | ACG | GAG | TTC | CCA | GAA | CAC | TGC | GAG | ACT | AAT | CTG | GAC | TCG | TCG | TGT | CCG | AGC | AGC | TCT | CCT | CCC | GTC | TCG | **450** |
| **121** | **G** | **G** | **F** | **N** | **K** | **F** | **Q** | **T** | **E** | **F** | **P** | **E** | **H** | **C** | **E** | **T** | **N** | **L** | **D** | **S** | **S** | **C** | **P** | **S** | **S** | **S** | **P** | **P** | **V** | **S** | **150** |
| **451** | GTT | CTG | GGT | CTG | AGC | GGA | TTA | AAG | ATC | AGC | TCC | GAC | GGA | TCG | GAC | GGT | GAA | TCG | GAC | CGG | GAG | CCG | AGC | AGC | GCC | ACC | GAG | TCG | GAC | GGC | **540** |
| **151** | **V** | **L** | **G** | **L** | **S** | **G** | **L** | **K** | **I** | **S** | **S** | **D** | **G** | **S** | **D** | **G** | **E** | **S** | **D** | **R** | **E** | **P** | **S** | **S** | **A** | **T** | **E** | **S** | **D** | **G** | **180** |
| **541** | AGC | CCG | TTG | CCT | AGC | AAC | CAG | CCG | GCG | TTC | CCG | GTC | CAG | ATC | CTG | CCC | TAT | TTG | TAT | TTA | GGC | TGC | GCT | AAA | GAC | TCC | ACC | AAC | CTG | GAC | **630** |
| **181** | **S** | **P** | **L** | **P** | **S** | **N** | **Q** | **P** | **A** | **F** | **P** | **V** | **Q** | **I** | **L** | **P** | **Y** | **L** | **Y** | **L** | **G** | **C** | **A** | **K** | **D** | **S** | **T** | **N** | **L** | **D** | **210** |
| **631** | GTC | CTC | GGA | AAA | TAC | AAC | ATC | AAG | TAC | ATT | CTG | AAC | GTG | ACG | CCC | AAC | CTG | CCC | AAC | ATG | TTC | GAG | CAT | GAC | GGC | GAA | TTC | AAG | TAC | AAG | **720** |
| **211** | **V** | **L** | **G** | **K** | **Y** | **N** | **I** | **K** | **Y** | **I** | **L** | **N** | **V** | **T** | **P** | **N** | **L** | **P** | **N** | **M** | **F** | **E** | **H** | **D** | **G** | **E** | **F** | **K** | **Y** | **K** | **240** |
| **721** | CAG | ATT | CCC | ATC | TCG | GAT | CAC | TGG | AGC | CAG | AAC | CTG | TCG | CAG | TTT | TTC | CCA | GAG | GCC | ATT | TCC | TTC | ATC | GAT | GAA | GCT | CGC | TCT | AAG | AAG | **810** |
| **241** | **Q** | **I** | **P** | **I** | **S** | **D** | **H** | **W** | **S** | **Q** | **N** | **L** | **S** | **Q** | **F** | **F** | **P** | **E** | **A** | **I** | **S** | **F** | **I** | **D** | **E** | **A** | **R** | **S** | **K** | **K** | **270** |
| **811** | TGT | GGT | ATC | CTG | GTG | CAC | TGC | CTG | GCG | GGG | ATT | AGT | CGC | TCG | GTC | ACG | GTG | ACG | GTG | GCG | TAT | CTG | ATG | CAG | AAA | CTC | AAC | CTG | AGT | CTA | **900** |
| **271** | **C** | **G** | **I** | **L** | **V** | **H** | **C** | **L** | **A** | **G** | **I** | **S** | **R** | **S** | **V** | **T** | **V** | **T** | **V** | **A** | **Y** | **L** | **M** | **Q** | **K** | **L** | **N** | **L** | **S** | **L** | **300** |
| **901** | AAT | GAC | GCT | TAC | GAC | TTC | GTC | AAG | CGC | AAA | AAA | TCG | AAC | ATC | TCA | CCC | AAC | TTT | AAC | TTC | ATG | GGG | CAG | CTG | CTG | GAC | TTC | GAG | CGA | ACT | **990** |
| **301** | **N** | **D** | **A** | **Y** | **D** | **F** | **V** | **K** | **R** | **K** | **K** | **S** | **N** | **I** | **S** | **P** | **N** | **F** | **N** | **F** | **M** | **G** | **Q** | **L** | **L** | **D** | **F** | **E** | **R** | **T** | **330** |
| **991** | CTG | GGG | TTA | AAC | AGT | CCG | ACC | TGC | GAC | AAC | CGG | TCG | CCC | AAA | GAG | CAG | CTG | TTC | TTC | ACC | ACA | CCC | ACC | AAT | CAC | AAC | GTG | TTC | CAG | CTC | **1080** |
| **331** | **L** | **G** | **L** | **N** | **S** | **P** | **T** | **C** | **D** | **N** | **R** | **S** | **P** | **K** | **E** | **Q** | **L** | **F** | **F** | **T** | **T** | **P** | **T** | **N** | **H** | **N** | **V** | **F** | **Q** | **L** | **360** |
| **1081** | GAT | ACG | CTC | GAG | TCA | ACG | TGA |  |  |  |  |  |  |  |  |  |  |  |  |  |  |  |  |  |  |  |  |  |  |  | **1170** |
| **361** | **D** | **T** | **L** | **E** | **S** | **T** | **.** |  |  |  |  |  |  |  |  |  |  |  |  |  |  |  |  |  |  |  |  |  |  |  | **390** |

DUSP10

| **1** | ATG | CCT | CCA | GCC | TCC | CTC | GAT | GAC | AGA | TTT | GTG | GTG | CCG | CTC | CAA | TTT | TAT | CTG | GAC | ACA | ACC | TAC | CTG | GAA | GCC | ACC | GTG | GGC | ACC | ATT | **90** |
| --- | --- | --- | --- | --- | --- | --- | --- | --- | --- | --- | --- | --- | --- | --- | --- | --- | --- | --- | --- | --- | --- | --- | --- | --- | --- | --- | --- | --- | --- | --- | --- |
| **1** | **M** | **P** | **P** | **A** | **S** | **L** | **D** | **D** | **R** | **F** | **V** | **V** | **P** | **L** | **Q** | **F** | **Y** | **L** | **D** | **T** | **T** | **Y** | **L** | **E** | **A** | **T** | **V** | **G** | **T** | **I** | **30** |
| **91** | GTG | GTG | GAG | ATC | CAG | GTG | ACC | AAC | CTT | ATT | TAT | ATG | CCC | TCA | TCC | AGC | AGC | TCT | ACC | CGT | TCC | CTT | ACG | TGT | GGA | TGC | AAC | ACT | GCC | AGC | **180** |
| **31** | **V** | **V** | **E** | **I** | **Q** | **V** | **T** | **N** | **L** | **I** | **Y** | **M** | **P** | **S** | **S** | **S** | **S** | **S** | **T** | **R** | **S** | **L** | **T** | **C** | **G** | **C** | **N** | **T** | **A** | **S** | **60** |
| **181** | TGT | TGC | ACG | GTG | AGC | ACC | TAC | GAA | AAA | GAC | AGC | CAG | ACA | CAG | ACC | CTG | ACC | CAA | ACA | CAG | AGC | CAG | GTC | AGT | ACC | AGT | AGC | CCC | AAC | TTG | **270** |
| **61** | **C** | **C** | **T** | **V** | **S** | **T** | **Y** | **E** | **K** | **D** | **S** | **Q** | **T** | **Q** | **T** | **L** | **T** | **Q** | **T** | **Q** | **S** | **Q** | **V** | **S** | **T** | **S** | **S** | **P** | **N** | **L** | **90** |
| **271** | AAT | TCC | GGA | GTG | AAC | TAT | GGT | GGG | CAA | GGA | GGT | TTC | TCC | AGG | CCG | ACG | GTG | GGC | CAG | AGT | GAA | ACC | TAC | AGT | GCA | CCC | AGT | CTC | ACT | TCC | **360** |
| **91** | **N** | **S** | **G** | **V** | **N** | **Y** | **G** | **G** | **Q** | **G** | **G** | **F** | **S** | **R** | **P** | **T** | **V** | **G** | **Q** | **S** | **E** | **T** | **Y** | **S** | **A** | **P** | **S** | **L** | **T** | **S** | **120** |
| **361** | GCC | ACA | CCC | AGA | GGT | GGT | GTG | CGT | ATC | ATC | CAC | CAG | AAT | GAG | CTG | GCT | CAG | AAG | ATG | ACA | CAT | TAT | CTC | ACA | GGT | CAT | CCA | GTA | GGA | CCT | **450** |
| **121** | **A** | **T** | **P** | **R** | **G** | **G** | **V** | **R** | **I** | **I** | **H** | **Q** | **N** | **E** | **L** | **A** | **Q** | **K** | **M** | **T** | **H** | **Y** | **L** | **T** | **G** | **H** | **P** | **V** | **G** | **P** | **150** |
| **451** | CTG | CCG | ATG | ATA | ATA | GAC | TGT | AGA | CCC | TTT | ATG | GAC | TAC | AAC | AAG | AGC | CAT | ATC | CGT | GGT | GCT | GTG | CAT | ATT | AAC | TGC | TCA | GAC | AAG | ATC | **540** |
| **151** | **L** | **P** | **M** | **I** | **I** | **D** | **C** | **R** | **P** | **F** | **M** | **D** | **Y** | **N** | **K** | **S** | **H** | **I** | **R** | **G** | **A** | **V** | **H** | **I** | **N** | **C** | **S** | **D** | **K** | **I** | **180** |
| **541** | AGC | CGC | CGG | CGT | CTG | CAG | CAG | GGC | AAG | ATC | ACT | GTG | CTG | GAT | CTC | ATT | TCC | TCC | TAC | CTG | AGC | AGA | GAC | TCA | TTT | AGG | GGG | ATT | TTT | TCT | **630** |
| **181** | **S** | **R** | **R** | **R** | **L** | **Q** | **Q** | **G** | **K** | **I** | **T** | **V** | **L** | **D** | **L** | **I** | **S** | **S** | **Y** | **L** | **S** | **R** | **D** | **S** | **F** | **R** | **G** | **I** | **F** | **S** | **210** |
| **631** | AAA | GAG | ATC | ATT | ATT | TAT | GAT | GAG | AGG | ACA | CAG | GAC | CCA | GCA | CGA | CTT | TCA | TCC | TCT | CAA | CCT | CTG | AGT | ATA | GTT | CTG | GAG | TCT | CTA | CAC | **720** |
| **211** | **K** | **E** | **I** | **I** | **I** | **Y** | **D** | **E** | **R** | **T** | **Q** | **D** | **P** | **A** | **R** | **L** | **S** | **S** | **S** | **Q** | **P** | **L** | **S** | **I** | **V** | **L** | **E** | **S** | **L** | **H** | **240** |
| **721** | AGG | GAT | GGG | AGG | GAC | CCA | ATG | GTT | CTC | CAA | GGT | GGC | ATT | TCC | AGT | TTC | AGA | CAG | ACC | TAT | GAG | GAC | TTA | TGT | GAG | GAC | TCC | CTA | CAA | CTT | **810** |
| **241** | **R** | **D** | **G** | **R** | **D** | **P** | **M** | **V** | **L** | **Q** | **G** | **G** | **I** | **S** | **S** | **F** | **R** | **Q** | **T** | **Y** | **E** | **D** | **L** | **C** | **E** | **D** | **S** | **L** | **Q** | **L** | **270** |
| **811** | CAG | GAG | GGT | CAC | GAT | GGT | GGT | GCA | GCT | GTT | GCT | CTT | TCG | GGG | GCA | CTA | CCT | CAC | ACG | CTG | CCC | TCC | AGC | CCT | GAC | ATT | GAG | AAT | GCT | GAG | **900** |
| **271** | **Q** | **E** | **G** | **H** | **D** | **G** | **G** | **A** | **A** | **V** | **A** | **L** | **S** | **G** | **A** | **L** | **P** | **H** | **T** | **L** | **P** | **S** | **S** | **P** | **D** | **I** | **E** | **N** | **A** | **E** | **300** |
| **901** | CTA | ACA | ACT | ATT | CTG | CCC | TTC | TTG | TAC | CTG | GGC | AAT | GAG | CAT | GAT | GCA | CAG | GAC | CTT | GAC | CAA | ATG | CAA | AGG | TTG | AAC | ATT | GGA | TAC | ATT | **990** |
| **301** | **L** | **T** | **T** | **I** | **L** | **P** | **F** | **L** | **Y** | **L** | **G** | **N** | **E** | **H** | **D** | **A** | **Q** | **D** | **L** | **D** | **Q** | **M** | **Q** | **R** | **L** | **N** | **I** | **G** | **Y** | **I** | **330** |
| **991** | CTC | AAT | GTT | ACC | ACA | CAC | CTC | CCA | CTA | TAC | CTC | TAT | GAC | CTT | GGG | ATG | TTC | AAG | TAT | AAA | CGC | TTA | CCT | GCC | ACC | GAC | AGC | AAC | AAG | CAG | **1080** |
| **331** | **L** | **N** | **V** | **T** | **T** | **H** | **L** | **P** | **L** | **Y** | **L** | **Y** | **D** | **L** | **G** | **M** | **F** | **K** | **Y** | **K** | **R** | **L** | **P** | **A** | **T** | **D** | **S** | **N** | **K** | **Q** | **360** |
| **1081** | AAC | CTA | CGT | CAG | TAC | TTT | GAA | GAA | GCA | TTT | GAA | TTT | ATA | GAA | GAA | GCT | CAC | CAG | GCA | GGC | AAG | GGA | CTG | CTC | ATT | CAC | TGC | CAG | GCA | GGT | **1170** |
| **361** | **N** | **L** | **R** | **Q** | **Y** | **F** | **E** | **E** | **A** | **F** | **E** | **F** | **I** | **E** | **E** | **A** | **H** | **Q** | **A** | **G** | **K** | **G** | **L** | **L** | **I** | **H** | **C** | **Q** | **A** | **G** | **390** |
| **1171** | GTG | TCC | CGG | TCT | GCC | ACT | ATT | GTC | ATT | GCG | TAC | CTG | ATG | AAA | CAC | ACT | TGG | ATG | ACC | ATG | ACA | GAT | GCC | TAC | AAG | TTT | GTC | AAG | ATG | CGA | **1260** |
| **391** | **V** | **S** | **R** | **S** | **A** | **T** | **I** | **V** | **I** | **A** | **Y** | **L** | **M** | **K** | **H** | **T** | **W** | **M** | **T** | **M** | **T** | **D** | **A** | **Y** | **K** | **F** | **V** | **K** | **M** | **R** | **420** |
| **1261** | AGG | CCG | ATC | ATC | TCG | CCC | AAC | CTC | ACC | TTC | ATG | GGC | CAG | CTC | CTT | GAG | TTT | GAA | GAA | GAT | CTC | AAT | AAT | GGA | ATC | ACT | CCT | CGC | ATA | CTC | **1350** |
| **421** | **R** | **P** | **I** | **I** | **S** | **P** | **N** | **L** | **T** | **F** | **M** | **G** | **Q** | **L** | **L** | **E** | **F** | **E** | **E** | **D** | **L** | **N** | **N** | **G** | **I** | **T** | **P** | **R** | **I** | **L** | **450** |
| **1351** | ACG | CCC | AAG | CTG | ATC | GGA | GTA | GAG | ACC | GTT | GTA | TAG |  |  |  |  |  |  |  |  |  |  |  |  |  |  |  |  |  |  | **1440** |
| **451** | **T** | **P** | **K** | **L** | **I** | **G** | **V** | **E** | **T** | **V** | **V** | **.** |  |  |  |  |  |  |  |  |  |  |  |  |  |  |  |  |  |  | **480** |
